# KIAA0556 is a novel ciliary basal body component mutated in Joubert syndrome

**DOI:** 10.1186/s13059-015-0858-z

**Published:** 2015-12-29

**Authors:** Anna A. W. M. Sanders, Erik de Vrieze, Anas M. Alazami, Fatema Alzahrani, Erik B. Malarkey, Nasrin Sorusch, Lars Tebbe, Stefanie Kuhns, Teunis J. P. van Dam, Amal Alhashem, Brahim Tabarki, Qianhao Lu, Nils J. Lambacher, Julie E. Kennedy, Rachel V. Bowie, Lisette Hetterschijt, Sylvia van Beersum, Jeroen van Reeuwijk, Karsten Boldt, Hannie Kremer, Robert A. Kesterson, Dorota Monies, Mohamed Abouelhoda, Ronald Roepman, Martijn H. Huynen, Marius Ueffing, Rob B. Russell, Uwe Wolfrum, Bradley K. Yoder, Erwin van Wijk, Fowzan S. Alkuraya, Oliver E. Blacque

**Affiliations:** School of Biomolecular and Biomedical Science, University College Dublin, Belfield, Dublin 4 Ireland; Department of Otorhinolaryngology, Radboud University Medical Center, PO Box 9101, 6500 HB Nijmegen, The Netherlands; Radboud Institute for Molecular Life Sciences, Radboud University Nijmegen, Nijmegen, The Netherlands; Department of Genetics, King Faisal Specialist Hospital and Research Center, Riyadh, Saudi Arabia; Department of Cell, Developmental, and Integrative Biology, University of Alabama at Birmingham Medical School, Birmingham, AL 35294 USA; Cell and Matrix Biology, Institute of Zoology, Focus Program Translational Neurosciences (FTN), Johannes Gutenberg University of Mainz, 55122 Mainz, Germany; Centre for Molecular and Biomolecular Informatics, Radboud Institute for Molecular Life Sciences, Radboud University Nijmegen, Nijmegen, The Netherlands; Department of Pediatrics, Prince Sultan Military Medical City, Riyadh, Saudi Arabia; CellNetworks, Bioquant, University of Heidelberg, Im Neuenheimer Feld 267, 69118 Heidelberg, Germany; Biochemie Zentrum Heidelberg (BZH), Im Neuenheimer Feld 328, 69120 Heidelberg, Germany; Department of Human Genetics, Radboud University Medical Center, PO Box 9101, 6500 HB Nijmegen, The Netherlands; Institute for Ophthalmic Research and Medical Proteome Center, Centre for Ophthalmology, Eberhard Karls University, Tuebingen, Germany; Department of Genetics, University of Alabama at Birmingham Medical School, Birmingham, AL 35294 USA; Department of Anatomy and Cell Biology, College of Medicine, Alfaisal University, Riyadh, Saudi Arabia

**Keywords:** Joubert syndrome, Cilia, KIAA0556, K04F10.2, Microtubule, Katanin, Basal body

## Abstract

**Background:**

Joubert syndrome (JBTS) and related disorders are defined by cerebellar malformation (molar tooth sign), together with neurological symptoms of variable expressivity. The ciliary basis of Joubert syndrome related disorders frequently extends the phenotype to tissues such as the eye, kidney, skeleton and craniofacial structures.

**Results:**

Using autozygome and exome analyses, we identified a null mutation in *KIAA0556* in a multiplex consanguineous family with hallmark features of mild Joubert syndrome. Patient-derived fibroblasts displayed reduced ciliogenesis potential and abnormally elongated cilia. Investigation of disease pathophysiology revealed that *Kiaa0556*^-/-^ null mice possess a Joubert syndrome-associated brain-restricted phenotype. Functional studies in *Caenorhabditis elegans* nematodes and cultured human cells support a conserved ciliary role for KIAA0556 linked to microtubule regulation. First, nematode KIAA0556 is expressed almost exclusively in ciliated cells, and the worm and human KIAA0556 proteins are enriched at the ciliary base. Second, *C. elegans* KIAA0056 regulates ciliary A-tubule number and genetically interacts with an *ARL13B* (*JBTS8*) orthologue to control cilium integrity. Third, human KIAA0556 binds to microtubules in vitro and appears to stabilise microtubule networks when overexpressed. Finally, human KIAA0556 biochemically interacts with ciliary proteins and p60/p80 katanins. The latter form a microtubule-severing enzyme complex that regulates microtubule dynamics as well as ciliary functions.

**Conclusions:**

We have identified KIAA0556 as a novel microtubule-associated ciliary base protein mutated in Joubert syndrome. Consistent with the mild patient phenotype, our nematode, mice and human cell data support the notion that KIAA0556 has a relatively subtle and variable cilia-related function, which we propose is related to microtubule regulation.

**Electronic supplementary material:**

The online version of this article (doi:10.1186/s13059-015-0858-z) contains supplementary material, which is available to authorized users.

## Background

Cilia are microtubule (MT)-based structures extending from the surface of most eukaryotic cells, serving important roles in cell and fluid motility, and sensory perception. These organelles also act as critical signalling hubs during development, transducing cues from ligands involved in cell–cell communication such as sonic hedgehog (Shh), platelet-derived growth factor and Wingless [1]. Defects in cilia underlie a wide range of human disorders, collectively called ciliopathies, characterised by a multitude of symptoms including cystic kidneys, retinal dystrophy, organ laterality defects, skeletal abnormalities, and peripheral and central nervous system defects [2]. One such disorder is Joubert syndrome (JBTS), a recessively inherited disorder affecting ~1/100,000 live births defined by hypotonia, ataxia, developmental delay, intellectual disability, episodes of neonatal fast or slow breathing, and dysmorphic facial features [3]. The hallmark feature of JBTS is a magnetic resonance imaging (MRI)-defined midbrain abnormality termed the ‘molar tooth sign’, resulting from cerebellar vermis hypoplasia, elongation and thickening of the superior cerebellar peduncles, and a deep interpeduncular fossa [3]. JBTS is often associated with wider ciliopathy symptoms such as polydactyly, kidney disease, retinal dystrophy, liver disease and endocrine problems. Thus, JBTS and related disorders overlap with other syndromic ciliopathies such as Meckel Gruber syndrome (MKS), Bardet-Biedl syndrome, nephronophthisis (NPHP) and oral-facial digital syndrome (OFD).

Most cilia are anchored by a mother centriole-derived basal body, consisting of a cartwheel arrangement of nine triplet (A, B, C) MTs with stabilising distal and subdistal appendages. The A and B tubules extend to form the characteristic nine doublet MT ring of the ciliary axoneme. The proximal-most portion of the axoneme, termed the transition zone, is defined by Y-link structures connecting each MT doublet to the ciliary membrane. These ciliary base structures (basal body and transition zone) are critical for cilium formation and function, and represent sites of action for many JBTS and other ciliopathy-associated proteins (e.g., OFD, MKS, NPHP). These actions include centriole migration, ciliary vesicle formation, subdistal and distal appendage assembly, and basal body docking [4–9]. The ciliary base, and in particular the distal appendages of the basal body centriole, is also a docking site for intraflagellar transport (IFT) machinery, which builds and maintains cilia by transporting various cargos into and out of the organelle [10, 11]. At the transition zone, multiple JBTS, MKS and NPHP proteins regulate ciliogenesis and the establishment of cytosolic and membrane diffusion barriers that regulate ciliary composition [12–16].

A number of ciliopathy-associated proteins, including JBTS proteins, regulate MT biogenesis, stability and post translational modification. Basal body-localised CEP41, linked to JBTS, regulates tubulin glutamylation [6]. OFD-associated C2CD3 at the distal ends of centrioles promotes centriole elongation, and ARL13B (JBTS8) and NPHP-4 regulate ciliary B-tubule integrity and A-tubule attachment [17–19]. KIF7, a mediator of Shh signalling and associated with hydrolethalus syndrome, binds to MT plus ends and regulates MT catastrophe and B-tubule integrity at the ciliary tip [20]. Recently, a centrosome-localised katanin p80 protein (KATNB1) associated with MT severing and mutated in primary microcephaly was shown to negatively regulate centriole and motile cilium formation [21, 22].

Here we identify a null mutation in *KIAA0556* in a JBTS family with an unusual additional pituitary involvement. Association with a relatively mild classic form of the disease correlates with a mouse *Kiaa0556* knockout model, which possesses a phenotype restricted to the brain. Investigation of the function of this uncharacterised gene in *Caenorhabditis elegans* roundworms and cultured human cells determined that KIAA0556 is a conserved basal body and MT-associated protein that genetically interacts with *ARL13B* (*JBTS8*), and biochemically associates with multiple ciliary proteins. Furthermore, we provide evidence that KIAA0556 influences the stability of the MT network, perhaps via direct regulation of katanin MT severing proteins.

## Results

### *KIAA0556* is mutated in Joubert syndrome

**Fig. 1:**
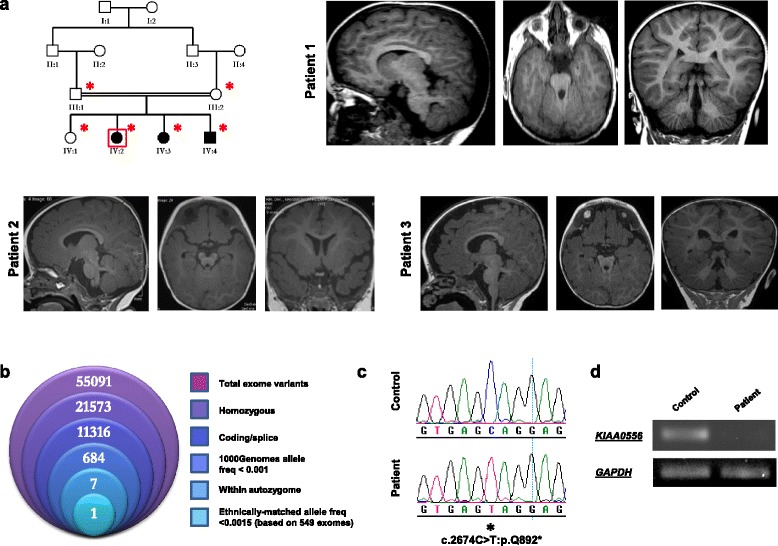
Identification of a *KIAA0556* nonsense mutation in a family with JBTS. **a** Pedigree of the multiplex consanguineous family with JBTS. The index (*boxed in red*) was submitted for exome capture, and the individuals denoted with *asterisks* were available for segregation testing. MRI cuts from patient 1 indicate ectopic posterior pituitary with severe hypoplasia/aplasia of anterior pituitary; vermis hypoplasia; superior cerebellar peduncle horizontal and thick with slightly deep interpeduncular fossa and enlarged prepontine cistern with increased vertical orientation of the brain stem. Patient 2 MRI reveals mild vermis hypoplasia; superior cerebellar peduncle horizontal; slightly deep interpeduncular fossa, and normal pituitary. MRI of patient 3 shows ectopic posterior pituitary with severe hypoplasia/aplasia of anterior pituitary; vermis hypoplasia; superior cerebellar peduncle horizontal and thick with dysmorphic mesencephalon; asymmetric cerebellar peduncle with flattened interpeduncular fossa and enlarged prepontine cistern with increased vertical orientation of the brain stem. **b** Filtering scheme of the exomic variants effectively narrowed the list of candidates to a single variant, KIAA0556:c.2674C > T:p.Q892*, the sequence chromatogram of which is shown in (**c**). **d** RT-PCR reveals near absence of the KIAA0556 transcript in patient cells compared with control

As part of our ongoing effort to characterise the genetic causes of ciliopathies, we examined a multiplex consanguineous Saudi Arabian family with three children suffering from global developmental delay and suspected JBTS based on neuroimaging studies (Fig. 1a). The first child is an 8-year-old girl whose neonatal history included transient tachypnea, hyperbilirubinema, hypotonia and recurrent upper respiratory tract infections. Global developmental delay became apparent later in infancy and a brain MRI revealed hallmark JBTS features in the posterior fossa, as well as a hypoplastic pituitary (Fig. 1a). Endocrinological evaluation revealed central hypothyroidism and growth hormone deficiency leading to hormone replacement therapy. Salient findings upon physical examination included short stature (despite supplemented growth hormone), ptosis, nystagmus, frontal bossing, hypertelorism, anteverted nares and hypotonia. This child did not display digit, orofacial cleft, or kidney (renal ultrasound) defects. Her 5-year-old sister presented with a similar history of global developmental delay, recurrent infections and hypotonia. However, she also has a history of occasional convulsions despite normal EEG recordings. Brain MRIs revealed milder JBTS features compared with her sister, mainly comprising inferior vermis hypoplasia. There was no evidence of hypopituitarism, although she has a history of oculoplasty to correct severe ptosis and preserved vision. The youngest affected is a 2.5-year-old brother, born with cleft lip and palate and a small penis, and who required minimal respiratory support after birth due to transient tachypnea. Given the family history, he was evaluated early with brain MRI and found to have mild cerebellar involvement mainly in the form of vermian hypoplasia. Although pituitary morphology was grossly intact, he had clear evidence of panhypopituitarism and is receiving hormone replacement. Like his two affected sisters, he suffers from global developmental delay.

Given the consanguineous pedigree structure, exome sequencing data were filtered to focus on regions of autozygosity shared exclusively between the three affected individuals. After subjecting the exome capture data to all filters (Fig. 1b; see "Materials and methods") one variant remained. This was a homozygous mutation in *KIAA0556* that predicts premature truncation of the protein at its approximate midpoint (NM_015202.2:c.2674C > T; p.Q892*) (Fig. 1c). The variant was not present in 615 ethnically matched exomes, and was confirmed to fully segregate with the disease. RT-PCR analysis on a patient-derived lymphoblastoid cell line revealed near absence of the *KIAA0556* mutant transcript, likely due to nonsense-mediated decay, indicating the mutation is likely a null allele (Fig. 1d). None of the known JBTS disease genes map to the regions of autozygosity shared exclusively between the three affected members of the family. Furthermore, all known JBTS disease genes were fully covered by the exome sequencing and none contained variants with predicted pathogenicity.

**Fig. 2:**
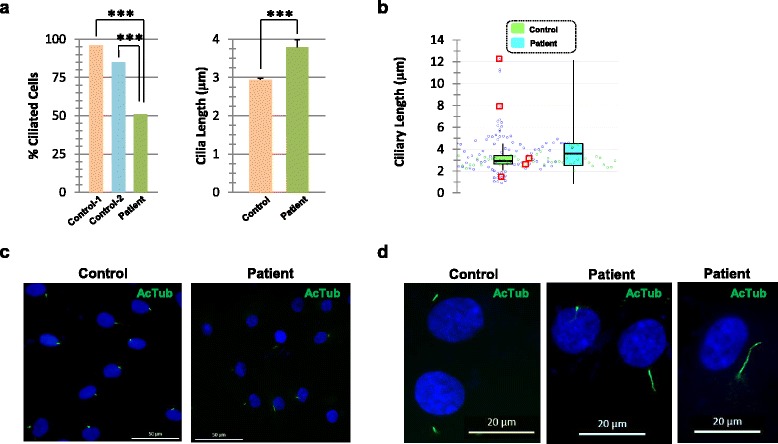
Patient-derived fibroblasts carrying a *KIAA0556* nonsense mutation show ciliogenesis defects. **a** Quantification of ciliogenesis potential and length of cilia shows significant reduction of ciliated cells and longer cilia in patient cells compared with control (***p < 0.0001 (two tailed t-test); 100 cells counted in each sample). Cilia were stained using an acetylated tubulin (AcTub) antibody. **b** Box and whisker plot indicating a much wider range of ciliary lengths in patient cells compared with control. Representative images showed decreased ciliogenesis potential (**c**) and increased ciliary length (**d**) in KIAA0556 cells from one of the patients

Since all known JBTS disease genes play a role in ciliary biology, ciliogenesis was examined in patient-derived fibroblast cells. Using a standard serum-starvation ciliogenesis assay, we assessed the potential of these cells to form cilia and observed significant reduction in the number of ciliated cells compared with controls (Fig. 2a, c). Interestingly, for those cells that were ciliated, the average cilium length was abnormally long (Fig. 2b, d).

Together, these data identify a likely null mutation in KIAA0556 causing JBTS in three siblings from a consanguineous family.

### *Kiaa0556* knockout mice develop hydrocephalus

**Fig. 3:**
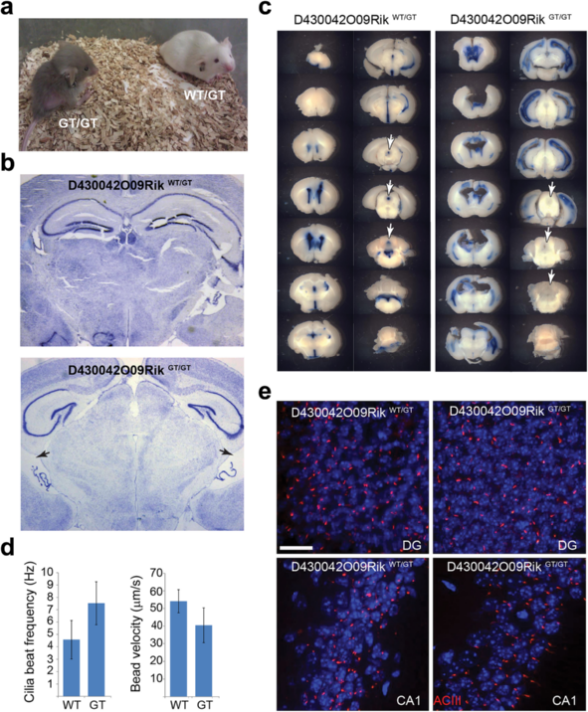
KIAA0556 mutation in mice results in hydrocephalus. **a** Mouse with D430042O09Rik genetrap (KIAA0556 mutation; *GT/GT*) with severe hydrocephalus next to heterozygous littermate (*WT/GT*). **b** Nissl stained brain sections from genetrap and heterozygous mice showing ventricle enlargement (*arrows*) and compressed hippocampi in the mutant. **c** Brain slices from mice injected with Evans blue to trace fluid flow through the ventricles. The genetrap mouse has blockage in the cerebral aqueduct (*arrows*) revealed by a lack of blue dye. **d** Analysis of the frequency of ependymal cilia beating and ventricular fluid flow velocity in genetrap (*GT*) and control wild-type (*WT*) mice showing similar rates. Error bars; standard deviations. **e** Sections of the hippocampus in genetrap and control mice showing normal primary cilia, indicated by adenyl cyclase III staining. Scale bar, 20 μm

To further assess the pathology associated with loss of KIAA0556 we generated a genetrap mutant mouse using the embryonic stem (ES) cell line D430042O09Rik^Gt(RRG309)Byg^ obtained from Bay Genomics. The genetrap integrated after exon 14 before entering the β-geo sequence in the genetrap integration vector. If translated, the 1610 amino acid KIAA0556 protein would be truncated at residue 600. When compared with littermates, homozygous recessive *Kiaa0556* knockout mice frequently displayed a hydrocephalus phenotype ranging from mild ventricular distension to severe skull malformation (Fig. 3a). Nissl-stained brain sections showed this hydrocephalus as an enlargement of the lateral ventricles and deformation of the hippocampus, indicating a defect in cerebral spinal fluid homeostasis (Fig. 3b). In heterozygous animals, injection of Evans blue dye into the lateral ventricle stained the entire ventricular system, whereas in the mutant brains the cerebral aqueduct and fourth ventricle showed no staining, indicating a form of non-communicating hydrocephalus due to blockage of the cerebral aqueduct (Fig. 3c). To determine if this hydrocephalus could be due to a defect in the ability of ependymal cilia to adequately move cerebral spinal fluid, we measured ependymal cilia beat frequency in the lateral ventricles using high speed video analysis. However, we found no significant differences in the rate of cilia motion between heterozygous and mutant animals (Fig. 3d). Since cilia seemed to be beating normally, we investigated cilium organisation and their ability to coordinate directed fluid flow in the knockout mouse. Specifically, the ventricles of acutely isolated brains were imaged whilst adding fluorescent beads to observe bulk fluid flow. Tracking the bead velocity indicated there was no appreciable difference in the ability of cilia to move cerebral spinal fluid in the mutant mice compared with the wild type (Fig. 3d). Furthermore, the distribution and number of primary cilia in sections of the CA1 and dentate gyrus (DG) appeared normal (Fig. 3e), and the kidney, liver, and lung tissue of these mutant animals was comparable to wild-type animals (data not shown). These data indicate that KIAA0556-disrupted mice possess brain-specific defects, resulting in a non-communicating (obstructive) hydrocephalus which does not appear to result from a gross defect in ventricular ependymal cilium structure or motility, although subtle effects on cilium ultrastructure or beating activity cannot be fully ruled out. Thus, the pathogenic mechanism underlying KIAA0556-associated hydrocephalus may not involve these motile cilia, similar to what is proposed for communicating hydrocephalus in mouse models of Bardet-Biedl syndrome [23].

### KIAA0556 is a conserved component of the ciliary base

KIAA0556 orthologues are found in most ciliated organisms, but missing from almost all non-ciliated organisms, indicating KIAA0556 is a conserved ciliary component since the last eukaryotic common ancestor (Additional file 1). All KIAA0556 orthologues possess three or four repeat sequences annotated as domains of unknown function in Pfam (DUF4457) (Additional file 1, boxed schematic). Interestingly, homology detection and structure prediction alignments (HHpred) [24] revealed that these repeats (~150–170 amino acids long) possess a remote sequence relationship with IFT25, which adopts a jelly-roll fold that interacts with the IFT27 GTPase [25]. Permutation of the repeat sequences by placing the beginning of each repeat (30–40 amino acids) to the end of the repeat improved the IFT25 alignments, suggesting a possible circular permutated relationship [26] between the KIAA0556 domains and IFT25 (Additional file 2).

**Fig. 4:**
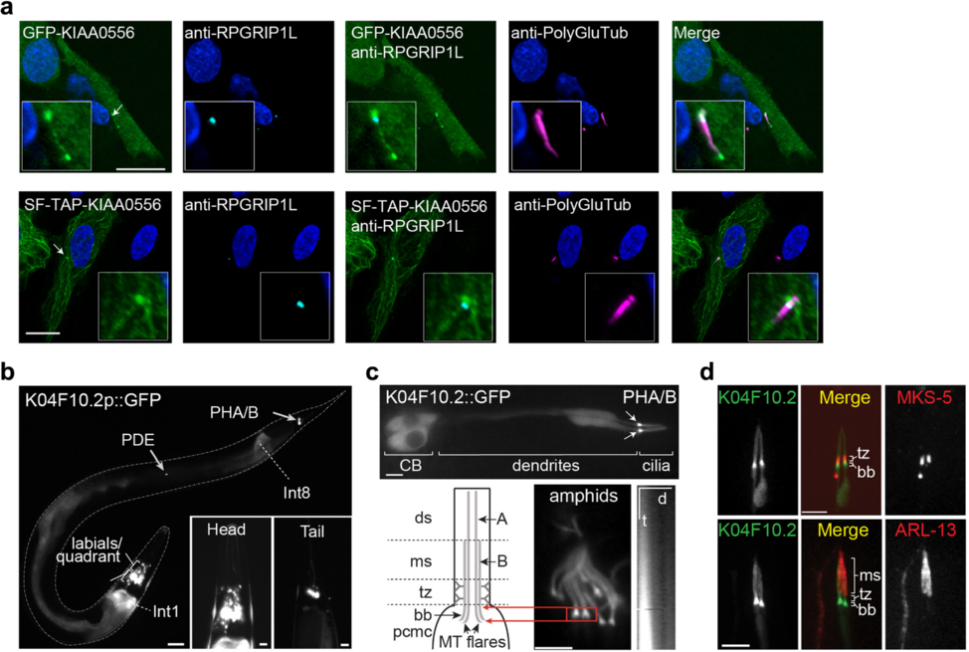
KIAA0556 is a basal body and MT-associated protein. **a** GFP- and Strep/FLAG tagged KIAA0556 is localised to the basal body of the primary cilium of RPE1 cells. Cells were counterstained for RPGRIP1L (*cyan*; transition zone marker) and polyglutamylated tubulin (PolyGluTub; *red*; ciliary and basal body marker). Scale bars, 20 μm. **b** A *C. elegans* KIAA0556 (K04F10.2) transcriptional reporter is expressed almost exclusively in ciliated cells (amphids, PHA/B phasmids, PDE, labial/quadrant neurons). Expression is also detected in two intestinal cells, Int1/Int8. Scale bars, 20 μm (whole worm), 5 μm (insets). **c, d** *C. elegans* K04F10.2::GFP accumulates at the ciliary base, proximal to the transition zone, and within the proximal ciliary axoneme (middle segment and transition zone). Schematic in (**c**) denotes the MT architecture of amphid and phasmid channel cilia, consisting of a distal segment (*ds*; singlet A tubules), middle segment (*ms*; doublet A/B tubules), transition zone (*tz*; containing Y-links) and proximal MT flares (analogous to a basal body; *bb*) in the periciliary membrane compartment (*pcmc*). The *red arrows* denote that the isosceles trapezoid shape of the K04F10.2::GFP ciliary base signal corresponds to that of the transmission electron microscopy-defined MT flares [29]. The kymograph (time over distance plot; *t*/*d*) in (c) is derived from a time lapse video and indicates that K04F10.2::GFP does not undergo processive movement in cilia. *White arrows* in (**c**) denote ciliary base accumulation of GFP signal. Fluorescence images in (**d**) captured from worms expressing K04F10.2::GFP and MKS-5::tdTomato transgenes show that a major pool of K04F10.2::GFP signal lies immediately proximal to TZ-localised MKS-5. *CB* cell body. Scale bars, 3 μm

To investigate if the KIAA0556 protein associates with cilia, we examined its subcellular localisation in human hTERT-RPE1 cells expressing green fluorescent protein (GFP) or Strep/FLAG (SF)-tagged constructs. In both cases, a specific pool of KIAA0556 was found at the ciliary base (Fig. 4a). In addition, GFP-tagged KIAA0556 decorated the ciliary axoneme, with frequent enrichment at the ciliary tip (Fig. 4a). In high expressing cells, both fusion proteins also localised to the cytoplasm where they appeared to decorate filamentous cytoskeletal structures (Fig. 4a).

To determine if KIAA0556 ciliary association is conserved, we investigated the *C. elegans* KIAA0556 orthologue K04F10.2*.* In *C. elegans*, cilia are found on 60 sensory head and tail neurons (out of a total of 959 cells in the adult hermaphrodite), located at the tips of long dendritic processes that terminate as sensory organs in the nematode cuticle [27]. Most sensory organs are found at the anterior (nose) end of the animal, and serve chemo-, osmo- and thermo-sensing functions [27]. To investigate in which cells of the worm the K04F10.2 gene is expressed, we analysed transgenic worms harbouring a GFP reporter under the control of the K04F10.2 promoter and found expression in many or most ciliated (neuronal) cells, with little or no expression in non-ciliated cells (Fig. 4b). A ciliated cell-specific expression pattern for K04F10.2 is essentially identical to that of many other ciliopathy gene orthologues, and correlates with a ciliogenic RFX transcription factor binding motif (X-box) in the K04F10.2 promoter [28]. To investigate the subcellular localisation of K04F10.2 in ciliated cells, we analysed transgenic worms expressing the entire K04F10.2 gene sequence fused to GFP. In agreement with the human KIAA0556 localisation, K04F10.2::GFP specifically accumulated at the ciliary base (Fig. 4c), immediately proximal to transition zone-localised MKS-5 (RPGRIP1L) (Fig. 4d). Weaker K04F10.2::GFP signals were also found in proximal regions of the ciliary axoneme, decorating the transition zone and the ARL-13 compartment of amphid and phasmid channel cilia (Fig. 4c, d). Interestingly, the ciliary base accumulation appeared as an isosceles trapezium, which could indicate that K04F10.2 associates with the proximal ends of the ciliary doublet MTs that flare out from the transition zone into the periciliary membrane compartment [29] (Fig. 4c, d). Although the precise roles of the MT flares are not known, they are thought to serve functions related to basal bodies, which are highly degenerate in *C. elegans* sensory cilia [29, 30]. Finally, using time-lapse microscopy, we found that K04F10.2::GFP does not undergo processive movement within the ciliary axonemes (e.g., IFT) or the neuronal processes (Fig. 4c, kymograph).

Together, these findings demonstrate that KIAA0556 is an evolutionarily conserved ciliary base protein, with additional localisations in the ciliary axoneme. Our data also suggest that KIAA0556 may associate with cytoskeletal proteins, or MTs in particular.

### KIAA0556 disrupted worms possess defects in ciliary MTs

**Fig. 5:**
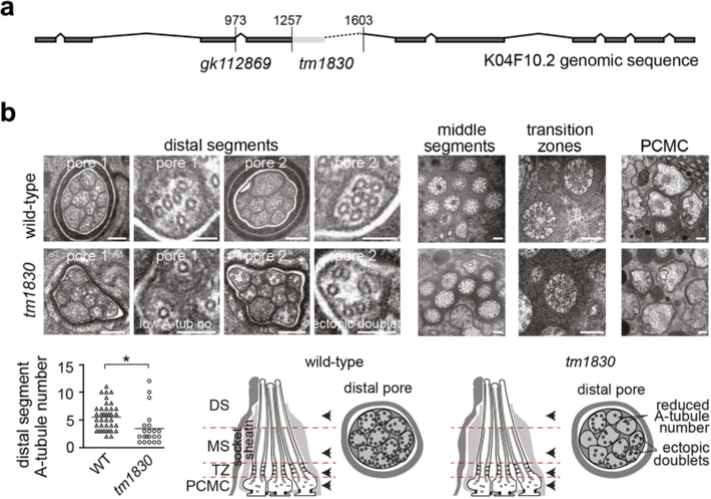
K04F10.2 mutant worms possess defects in ciliary MTs. **a** The K04F10.2 gene showing the *gk112869* nonsense mutation in exon 3 and the *tm1830* deletion (1257–1603) spanning exon 4 and intron 4. *Boxes* define exons. Numbers indicate genomic positions relative to the translational start codon. **b** Transmission electron microscopy (TEM) of amphid channel cilia in age-matched (early day 1 adult) wild-type (*WT*) and K04F10.2*(tm1810)* worms. Wild type channels (pores) contain ten ciliary axonemes, each possessing a distal segment (*DS*) containing singlet A-tubules, a middle segment (*MS*) containing doublet A/B tubules, a transition zone (*TZ*) with Y-link connectors, and a periciliary membrane compartment (*PCMC*). Images and graph show that *tm1810* mutants display a reduced number of distal segment outer singlet A-tubules; also, ectopic MT doublets are sometimes observed in the distal segment. Note that wild-type early day 1 adults do not possess a full complement of nine distal segment A-tubules. Schematics show the ultrastructural phenotypes (only three cilia shown for simplicity in longitudinal cartoons) and the *arrowheads* indicate approximate regions of pore where imaged sections were captured. Distal segment images shown at low (first and third images) and high (second and fourth images) magnifications. Scale bars, 200 nm (low magnification images), 100 nm (high magnification images). *p < 0.05 (t-test versus WT)

To further investigate the ciliary roles of KIAA0556, we assessed cilium structure and function in K04F10.2-disrupted worms. Two mutant alleles were employed, *tm1830* (347-bp frameshift deletion) and *gk112689* (nonsense point mutation), which if translated are predicted to produce severely truncated proteins of 208 and 132 amino acids, respectively (Fig. 5a). First, we used a dye uptake assay to indirectly assess the integrity of eight pairs of sensory cilia, six in the head (amphid cilia) and two in tail (phasmid cilia) [31], and found that both K04F10.2 mutants possess a normal dye-filling response, indicative of normal cilium structures (Additional file 3a). We confirmed that cilium structures were normal in *tm1830* mutants using GFP reporters that stain the rod-shaped ASER and PHA/B cilia, or the forked AWB cilium with membranous tip expansions (Additional file 3b). Next we assayed various cilia-related sensory functions and found that *tm1830* worms possess normal chemoattraction (to benzaldehyde), osmotic avoidance, and foraging behaviours (Additional file 3c–e). Finally, kymography-based assays were employed to investigate the ciliary transport (IFT) of GFP-tagged OSM-3 (KIF17), CHE-11 (IFT140) and OSM-6 (IFT52) along amphid and phasmid channel cilia. Anterograde and retrograde IFT rates were mostly unaffected in *tm1830* mutants, although there were small significant (*p* < 0.001) reductions (<10 %) in the anterograde rates of OSM-6 (along middle segments) and OSM-3 (along distal segments) (Additional file 4). Consistent with a mostly normal IFT system in these worms, all three markers displayed normal ciliary localisations and distributions (Additional file 4b).

Despite these grossly normal features of cilium structure, function and transport, ultrastructural analyses revealed significant MT defects in the amphid channel cilia of *tm1830* mutants. In wild-type worms, these cilia are segmented into a proximal region (transition zone and middle segment) consisting of nine outer doublet MTs, and a distal region (distal segment) consisting of outer singlet A-tubules due to the termination of each doublet B-tubule at the middle segment tips (Fig. 5b). In the cilia of *tm1830* mutants, age-matched with wild-type controls (early day 1 adult), a 40 % reduction in A-tubule outer singlet number was observed in distal segments; also, whilst many *tm1830* cilia retained a bipartite MT arrangement, MT doublets were occasionally observed at the ciliary tips (Fig. 5b).

Thus, loss of K04F10.2 disrupts ciliary MT load and organisation, although various aspects of gross cilium length, function and transport are mostly normal, at least in those cilia examined.

### *C. elegans* KIAA0556 genetically interacts with *ARL13B* (*JBTS8*)

The relatively subtle requirement of K04F10.2 in regulating ciliary MTs prompted us to investigate if this gene functionally interacts with ciliary transport or ciliopathy genes, as previously shown for various *C. elegans* IFT and ciliopathy genes, including those associated with JBTS [12, 16, 32–35]. Using double mutants, K04F10.2 was tested for genetic interactions with *klp-11* (*KIF3B*; kinesin-2 subunit), middle segment-localised *arl-13* (*ARL13B/JBTS8*), as well as transition zone-associated *mks-5* (*RPGRIP1L*) and *nphp-4* (*NPHP4*). These genes were selected on the basis of direct pathogenic associations with JBTS-related disorders (*arl-13* [36, 37], *mks-5* [38, 39]), or genetic interactions with JBTS genes (*nphp-4* [16, 40], *klp-11* [40]); also, the corresponding mutants retain significant cilium structure and function, thus allowing the possibility of observing synthetic phenotypes.

**Fig. 6:**
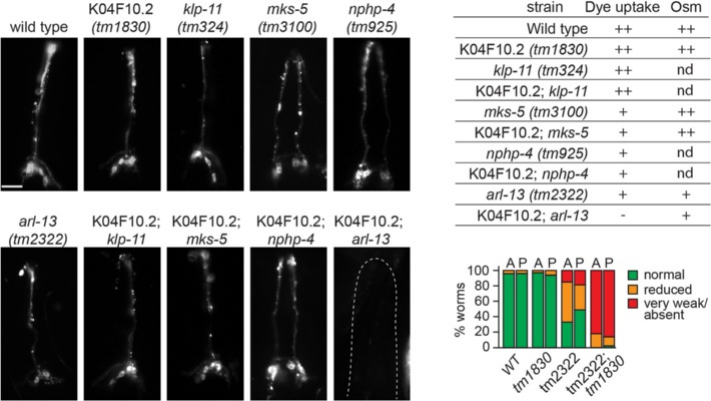
*C. elegan*s K04F10.2 genetically interacts with JBTS-associated *arl-13* to control dye-filling. Shown are fluorescence images of amphid (head) neurons following a dye-filling assay, which indirectly measures cilium integrity. Table shows the extent of the dye-filling phentoypes, scored as normal (++), reduced (+), or very weak/absent (-); *nd* not determined. The table also shows osmotic avoidance behaviours scored in the same way. The graph shows quantification of the dye filling phenotype for *arl-13(tm2322)* and K04F10.2(*tm1830*) single mutants, and the corresponding double mutant. *A* amphid neurons, *P* phasmid neurons, *WT* wild type. Scale bar, 15 μm

Using dye-filling and osmotic avoidance assays investigating cilium integrity and function, we found that double mutants of K04F10.2(*tm1830*) with *klp-11(tm324)*, *mks-5(tm3100)* or *nphp-4(tm925)* possess the same phenotypes as the corresponding single mutants (Fig. 6). However, a double mutant of *tm1830* together with a *tm2322* mutation in *arl-13* caused a synthetic dye-filling phenotype, with DiI uptake severely reduced or absent in head and tail neurons (Fig. 6). This synthetic interaction was not observed for osmotic avoidance, indicating this ASH cilium-mediated phenotype in *arl-13* mutants is not modified by K04F10.2 disruption. Thus, *arl-13* and K04F10.2 genetically interact to control dye-filling, and therefore cilium integrity, indicating a functional relationship for these two JBTS gene orthologues. Interestingly, mouse and worm Arl13b mutants possess defects in ciliary A/B tubule connections and polyglutamylation [18, 32, 35, 40]. Although these phenotypes differ from the KIAA0556 worm mutant (reduced A-tubule number), both genes are nonetheless required for the normal integrity of ciliary MTs, and this may explain the synthetic phenotype observed in the *C. elegans* double mutant.

### Human KIAA0556 binds to MTs and regulates MT stability

**Fig. 7:**
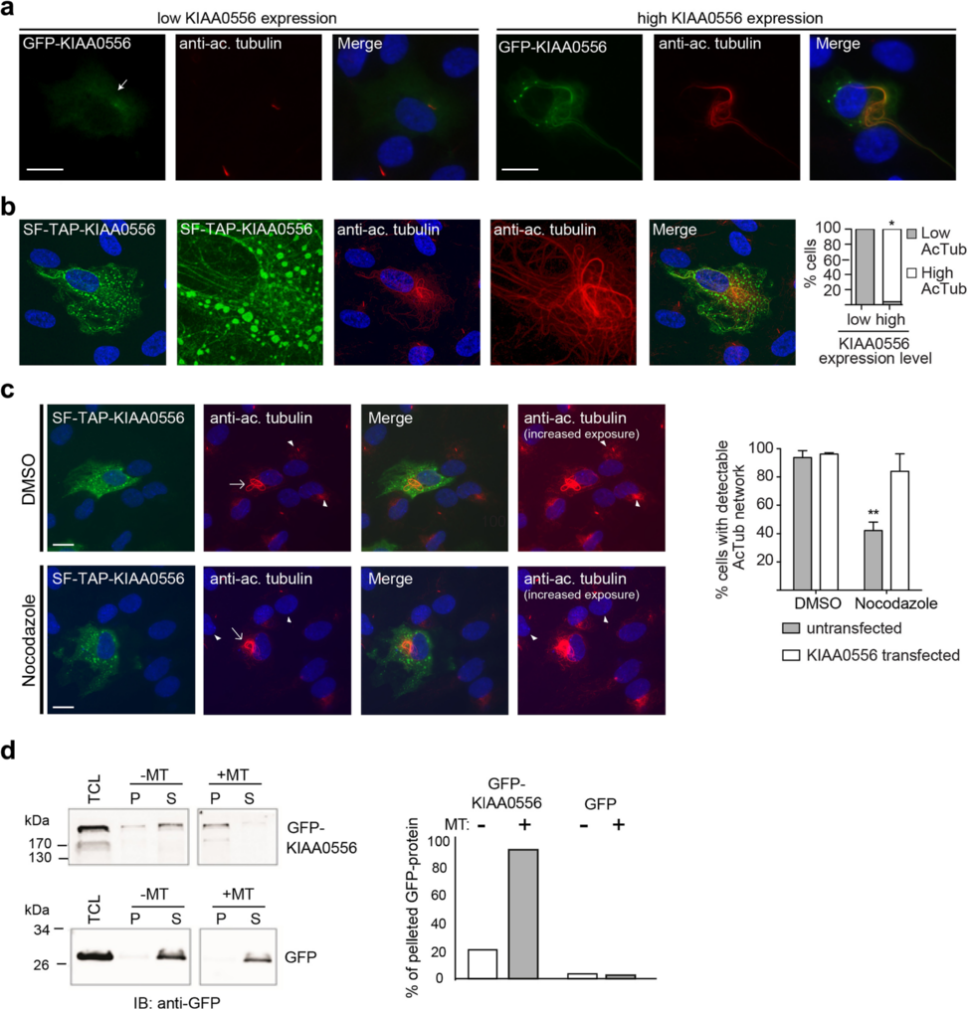
Human KIAA0556 binds to and stabilises MTs. **a** In hTERT-RPE1 cells with high GFP-KIAA0556 expression levels, counterstained for acetylated alpha-tubulin (*red*), KIAA0556 colocalises with MTs; also, acetylated tubulin levels are enhanced, indicating a stabilised MT network. This is not observed in cells expressing low levels of GFP-KIAA0556. Scale bars, 20 μM. **b** Similar features are observed for SF-TAP-tagged KIAA0556 (*green*). Shown is a representative example of a high expressing KIAA0556 cell (low expressing cell example not shown). Images were generated by confocal laser scanning and are presented as maximum projections, to rule out differences in focal depth. *p < 0.001 (compared with low KIAA0556 expression dataset; Fisher’s exact test; n = 63). **c** SF-TAP-KIAA0556 transfected hTERT-RPE1 cells are resistant to nocodazole-induced destabilisation of the MT network. Cells with high SF-TAP-KIAA0556 expression (*arrows*) display a stabilised MT network, characterised by high levels of acetylated alpha-tubulin staining (*red*, normal exposure images). In non-transfected cells (*arrowheads* in high-exposure panel), 10 minute nocodazole (10 μM) treatment destabilised the MT network. Although some acetylated tubulin immunoreactivity remained in these cells (e.g., in the cilium and close to the nucleus), a filamentous MT network pattern was not observed. In contrast, SF-TAP-KIAA0556 transfected cells retain a stabilised MT network after nocodazole treatment. Normal and increased exposure images show that a stabilised MT network (characterised by a filamentous acetylated tubulin staining pattern) is observed in almost all DMSO treated transfected and untransfected cells (*arrowheads*). Data in the graph (two experiments) are derived from the analysis of >1000 untransfected cells and >110 transfected cells per condition. Error bars; standard deviation of the mean. **p < 0.01 (one-way ANOVA). Scale bar, 20 μm. **d** KIAA0556 binds to microtubules in vitro probed by microtubule binding protein spin-down assays either with GFP-KIAA0556 (*upper panel*) or GFP (*lower panel*, negative control) expressed in HEK293 cells. In the absence of supplementary microtubules (*-MT*) KIAA0556 and GFP are found in the supernatant (*S*). In the presence of supplementary microtubules (*+MT*) KIAA0556 is predominantly recovered in the pellet (*P*). Quantification of band densities revealed 93 % of KIAA0556 in the microtubule pellet fractions. *TCL* 10 % of total cell lysate (input), *IB* immunoblot

To further investigate possible associations between KIAA0556 and MTs, we examined the MT network in hTERT-RPE1 cells overexpressing KIAA0556. In 95 % of cells expressing high levels of GFP- or SF-TAP-tagged KIAA0556, a more pronounced immunoreactivity against acetylated alpha tubulin was observed compared with cells expressing low levels of KIAA0556 (Fig. 7a, b). Co-stained cells also showed that a significant amount of the overexpressed KIAA0556 colocalised with acetylated alpha tubulin signals, thus explaining (at least in part) the filamentous cytoplasmic signals reported above in Fig. 4a.

Since acetylation stabilises MTs, the increased acetylated alpha tubulin signals we observed in cells with high levels of KIAA0556 expression suggest that cytoplasmic MTs might be hyperstabilised in these cells. To directly investigate this possibility we treated cells with the MT destabilizing agent nocodazole [41]. A ten minute incubation with nocodazole disrupted intracellular MT networks in ~60 % of untransfected cells, whereas MTs remained intact in most cells expressing high levels of SF-TAP-KIAA0556 (Fig. 7c; Additional file 5). Similar observations were made for cells expressing high levels of GFP-tagged KIAA0556 (Additional file 5). Thus, overexpression of KIAA0556 stabilises cytoplasmic MTs, and this finding is consistent with the increased levels of acetylated MTs we observe in these cells.

Finally, we investigated whether KIAA0556 biochemically associates with MTs in vitro using a MT binding protein spin-down assay. In this assay proteins binding to MTs are recovered after ultracentrifugation in the pellet together with the heavy MTs, whereas non-binding proteins stay in the supernatant. Whilst in the absence of supplementary MTs GFP-KIAA0556 and GFP were both primarily present in the supernatant, after adding MTs over 90 % of GFP-KIAA0556 was recovered in the pellet (Fig. 7d). Substantial recruitment of KIAA0556 to the pellet in the presence of supplementary MTs indicates KIAA0556 binds to MTs.

### Human KIAA0556 biochemically associates with ciliary components and katanins

**Fig. 8:**
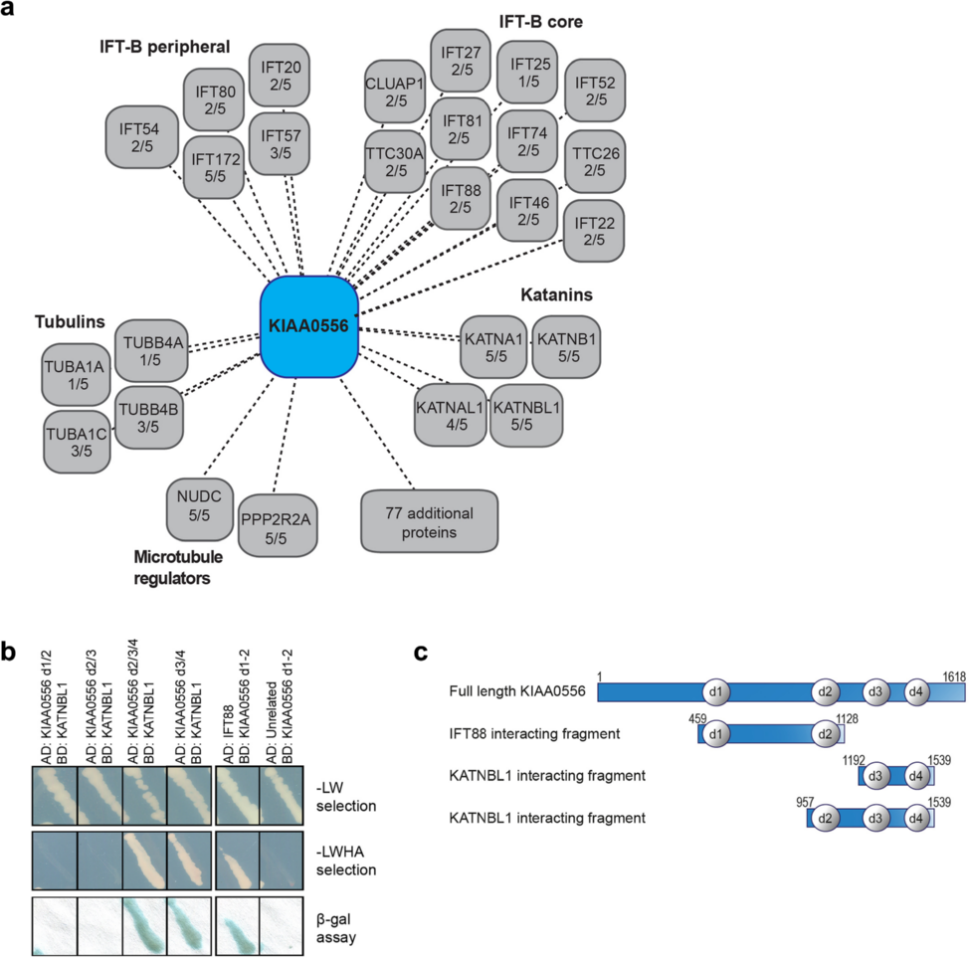
KIAA0556 is associated with several groups of ciliary proteins. **a** Visual representation of the preys identified in the TAP experiments. Proteins are clustered according to established protein complexes and the number of experiments in which each protein was identified. The full dataset is shown in Additional file 6. **b** Yeast two-hybrid (Y2H) experiment showing the interaction between fragments of the KIAA0556 protein (fused to the GAL4 activation domain; AD) and KATNBL1 and IFT88 (fused to the GAL4 DNA-binding domain; BD). An unrelated protein was used as negative control for KIAA0556 d1/2 LW; selective media lacking leucine, tryptophan. LWHA; selective media lacking leucine, tryptophan, histidine, adenine. The regions of KIAA0556 that interact with these proteins are depicted schematically in **c**. The predicted protein repeat domains are represented as d1 to d4. Additional file 7a shows all the KIAA0556 fragments tested in Y2H assays

To get a better insight into the molecular basis of KIAA0556 function, we used a tandem affinity purification (TAP) strategy followed by mass spectrometry to identify proteins that biochemically associate with human KIAA0556. KIAA0556 was N-terminally tagged with a Strep/FLAG tag (SF-TAP-tag), and expressed and isolated from human embryonic kidney cells (HEK293T) as described previously [42]. After excluding likely false positives (see "Materials and methods" section), a final list of 128 proteins (n = 5 experiments) was generated (Fig. 8a; Additional file 6). The most commonly identified prey proteins belong to a katanin module, which was co-purified and highly ranked in all five TAP experiments (Fig. 8a; Additional file 6). In total, four subunits of the katanin complex were identified: two p60 proteins (KATNA1, KATNAL1) and two p80 proteins (KATNB1, KATNBL1). KATNA1 (enzymatic) and KATNB1 (accessory) are thought to form a MT-severing enzyme complex that regulates MT mass, stability and elongation, as well as ciliary functions that include flagellar length control, stress-induced deciliation, MT central pair formation, centriole/cilia number and Shh signalling [21, 22, 43–47]. Ciliary roles have not been associated with the KATNA1/B1 paralogues, KATNAL1 and KATNBL1, although ciliogenic roles are reported for related KATNAL2 proteins that localise to cilia [48] (not present in our TAP datasets). Additional high ranking hits that form protein clusters were several tubulins and IFT-B proteins (two TAP experiments), with IFT172 found in all five TAP experiments (Fig. 8a; Additional file 6).

As a complementary approach, a dedicated one-on-one yeast two-hybrid approach was used to screen KIAA0556 fragments against a panel of ~200 cilium-associated proteins, as well as various katanin subunits (KATNA1, KATNAL2 and KATNBL1). Specifically, we employed four bait fragments, each containing two or three of the four repeat domains in KIAA0556 (Additional file 7a). In agreement with our TAP data, two of the baits (containing domains 3/4 or 2/3/4) showed a positive interaction with full length KATNBL1, indicating that KATNBL1 directly interacts with the C-terminal half of KIAA0556 (Fig. 8b, c). Positive test results were also obtained for KIAA0556 baits against IFT88 (Fig. 8b, c).

Together, these finding demonstrate that KIAA0556 is biochemically associated with ciliary proteins and with katanin subunits implicated in MT severing.

### Conserved localisation of KATNBL1 at the ciliary base region

**Fig. 9:**
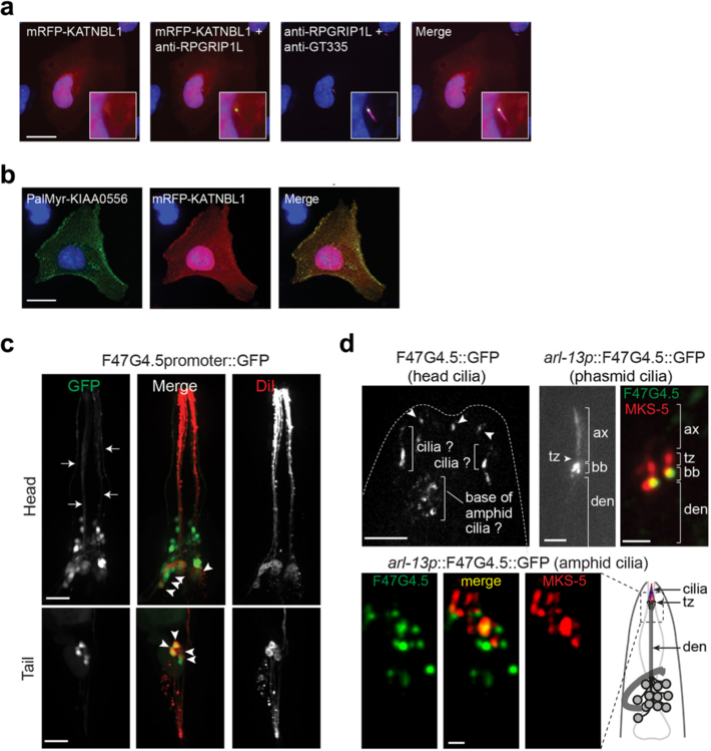
Conserved localisation of KATNBL1 to the ciliary base region. **a** mRFP-tagged KATNBL1 is enriched at the basal body and ciliary axoneme, as well as the nuclear membrane. Cells are counterstained for ARL13B (*magenta*). Scale bars, 20 μm. **b** PalMyr assay visualising the interaction between KIAA0556 and KATNBL1 in a cell system. The PalMyr tagged protein (*green*) is targeted towards the cell membrane. The interacting protein, tagged with mRFP (*red*), follows this induced localisation at the cell membrane, apparent from the overlay of both the green and red signals. Single transfected cells are shown in Additional file 6b. Scale bars, 20 μm. **c** The expression of a *C. elegans* KATNBL1 homologue, F47G4.5, is mostly restricted to ciliated cells. Shown are fluorescence images of worms expressing a transcriptional GFP reporter under the control of the F47G4.5 promoter, which stains the entire cell. DiI (*red*) co-stain identifies six pairs of ciliated amphid neurons in the head and both pairs of ciliated phasmid neurons in the tail. *Arrowheads* denote cells with both red and green signals. Other ciliated head cells are identifiable by long dendritic processes (*arrows*) extending to the anterior end of the worm. Scale bars, 20 μm. **d** The *C. elegans* F47G4.5 protein localises near the base of cilia and occasionally in the ciliary axonemes. Shown are fluorescent images of the anterior head (*top left image*), phasmid cilia (*top right images*) and amphid cilia regions of transgenic worms expressing F47G4.5::GFP alone (*top left* and *middle images*) or F47G4.5::GFP together with the ciliary transition zone marker MKS-5::tdTomato (*top right* and *bottom images*). Two colour images are maximum projections of a Z-stack series imaged on a spinning disk confocal microscope. *ax* ciliary axoneme, *tz* transition zone, *bb* basal body, *den* dendrite. The *dashed box* in the schematic denotes the imaged region in the adjacent panels. Scale bars, 5 μm (*top left image*), 2 μm (*all other images*)

We interrogated further the relationship between human KATNBL1 and KIAA0556 by examining the subcellular localisation of monomeric red fluorescent protein (mRFP)-tagged KATNBL1 expressed in hTERT-RPE1 cells. Like KIAA0556, we observed a pool of KATNBL1 protein at the ciliary base and along the ciliary axoneme (Fig. 9a). Furthermore, we found that a KIAA0556 construct (Pal-Myr tagged) ectopically targeted to the plasma membrane induced a similar ectopic localisation of KATNBL1, corroborating the biochemical interaction between these two proteins (Fig. 9b; Additional file 7b).

We also investigated if katanin subunits, including KATNBL1, are associated with cilia in *C. elegans*. The worm genome contains four katanin genes encoding homologues of p60 KATNA1 (MEI-1), canonical p80 KATNB1 (F47G4.4), and two paralogues of non-canonical (WD40-less) p80 KATNBL1 (MEI-2 and F47G4.5) [49]. Using GFP reporters under the control of katanin gene promoter sequences, we found that F47G4.5/KATNBL1 expression was mostly restricted to amphid and phasmid ciliated neurons, as well as several ciliated labial/quadrant neurons that send identifiable dendritic processes to the nematode nose tip (Fig. 9c). This expression pattern is very similar to that observed for K04F10.2 described above in Fig. 4b. In contrast, although *mei-1* was expressed in at least some ciliated head neurons, *mei-1*, *mei-2* and F47G4.4 were generally not expressed in many identifiable ciliated cells (Additional file 8). A restricted ciliated cell expression pattern for F47G4.5 agrees with the non-essential nature of this gene, compared with the more broadly expressed and essential *mei-1* and *mei-2* genes [50, 51].

Since F47G4.5 is specifically expressed in ciliated cells, we investigated the subcellular localisation of the encoded protein using an F47G4.5::GFP construct controlled by its own promoter. In transgenic worms expressing this construct, only very weak post-embryonic GFP signals were observed; however, these signals appeared to co-localise at or near the ciliary base or within the ciliary axonemes (Fig. 9d, upper left image). Because F47G4.5 expression may be downregulated post-embryonically, we made an F47G4.5::GFP construct under the control of the *arl-13* promoter, which is highly active in post-embyronic ciliated neurons [40, 52]. Using this construct we confirmed that F47G4.5 localises at the ciliary base region, proximal to transition zone-localised MKS-5, and occasionally within the ciliary axonemes of at least a few neurons (phasmids and various head cilia) (Fig. 9d). The F47G4.5::GFP signals at the ciliary base region were somewhat varied; in some images, the GFP signals bordered the more distal transition zone (MKS-5) signals, whereas in other images a small gap could be observed between the F47G4.5 and MKS-5 localisations.

Together, these data from human cells and *C. elegans* show that KATNBL1 and KIAA0556 possess overlapping ciliary localisation and expression properties. This is consistent with the biochemical interaction we observed for these proteins, and further supports a functional association between KIAA0556 and katanins in the modulation of MTs.

## Discussion

Our knowledge concerning the genetic heterogeneity of JBTS has greatly expanded over the past few years, due in large part to the high throughput of genomic sequencing tools especially when combined with positional mapping clues that are readily available in consanguineous pedigrees. Despite this significant progress, recent cohorts clearly show that not all disease genes in JBTS have been identified [53, 54]. In this study, we describe a multiplex consanguineous family in which the three affected siblings with JBTS do not map to any of the previously identified JBTS disease genes. Whole-exome sequencing in this family highlighted a nonsense mutation in *KIAA0556* as the only predicted pathogenic variant that is shared exclusively by the three affected siblings.

*KIAA0556*, as the generic name implies, is a poorly studied gene and was not known to be linked to any human disease. In order to support the notion that *KIAA0556* is a novel JBTS disease gene, we sought additional lines of evidence that link the *KIAA0556*-encoded protein to the primary cilium since this is where all previously reported JBTS genes are known to mediate disease pathogenesis. Structural ciliary defects were observed in patient cells that harbour the *KIAA0556* null mutation. Specifically, we observed significant reduction in the percentage of ciliated cells, a classic cellular phenotype of JBTS and other ciliopathies [55–59]. Surprisingly, we also observed abnormal lengthening of the cilia that did form, suggesting a negative regulatory role for KIAA0556 on ciliary length.

The results we obtained from *C. elegans* sensory neurons and cultured human cells corroborate a proposed ciliary function for KIAA0556. We show that the human KIAA0556 protein and its *C. elegans* orthologue are significantly enriched at the ciliary base, with additional signals in the ciliary axoneme. In addition, we show that *C. elegans* KIAA0556 genetically interacts with *arl-13*, the nematode orthologue of *ARL13B* (*JBTS8*), thereby placing KIAA0556 in a pathway with a known JBTS gene. Furthermore, TAP studies revealed that human KIAA0556 biochemically interacts with several known ciliary proteins, most notably intraflagellar transport components and katanins associated with MT severing. p60 and p80 katanins regulate ciliogenesis and MT central pair formation in protists, whereas in mammalian cells, KATNB1 (p80) inhibits ciliogenesis and is mutated in patients with microlissencephaly, which is a ciliopathy-related condition [21, 22, 43, 44]. The robust association of KIAA0556 with the katanin module in TAP experiments (5/5 experiments) is supported by our finding that human KIAA0556 directly interacts with KATNBL1 (KATNB1-like protein 1), and by the comparable ciliary base and axonemal localisations observed for these proteins in human and nematode cells. To our knowledge, this is the first report of a ciliary association for KATNBL1.

Independent of these results, we show that KIAA0556 deficiency in the mouse results in hydrocephalus, a known central nervous system ciliopathy phenotype associated with certain types of JBTS [60–62]. The limited phenotypic expression observed in this mouse line is consistent with the rather mild phenotype we observe in patients with a *KIAA0556* null mutation. It is also consistent with the many normal ciliary features observed in KIAA0556-disrupted worms. Indeed, we note that the three JBTS patients presented with a restricted phenotype (mild molar tooth sign), lacking the wider symptoms frequently associated with more severe forms of JBTS (eyes, kidneys and hands are apparently spared). Midline anomalies, including cleft lip/palate and single nostrils, have only been rarely observed in JBTS [57, 63]. The pituitary involvement in two of the three patients may represent a rare JBTS-associated midline anomaly and it will be interesting to observe the frequency of this in future patients with *KIAA0556*-related JBTS.

The mechanism by which KIAA0556 disruption exerts its pathogenic effect on cilia remains unclear. One possibility is that KIAA0556 regulates IFT, either the machinery or cargo. In support of an IFT association is sequence similarity to IFT25, biochemical evidence of an interaction between KIAA0556 and the IFT-B complex, a genetic interaction with the IFT regulator *arl-13* [32, 40], disrupted ciliogenesis in KIAA0556-deficient patient fibroblasts, and ciliary localisations. However, our data showing that nematode KIAA0556 does not undergo IFT, KIAA0556-disrupted worms and mice possess mostly normal cilium structures and IFT (worms), and human IFT-B complex members (with the exception of IFT172) were retrieved only in part of the TAP experiments argue against a broad role in regulating the IFT machinery. Nonetheless, in KIAA0556-disrupted worms, we did observe small reductions in anterograde IFT rates for OSM-6 along middle segments and OSM-3 along distal segments, although the respective distal (OSM-6) and middle (OSM-3) segment rates were normal, as were the middle and distal segment rates for CHE-11 (IFT140). Thus, it remains possible that KIAA0556 regulates the transport of a subset of IFT proteins along discrete segments of cilia, or ciliary subtypes. More detailed IFT rate analyses, including measurements from individual ciliary axonemes, will be required to further test this hypothesis. Alternatively, KIAA0556 may regulate specific cargo associations with IFT complexes at the ciliary base, which is the major site of KIAA0556 localisation. Tubulin, which biochemically interacts with IFT-B complexes [64], is an attractive candidate cargo for KIAA0556 regulation given our biochemical and co-localisation data implicating associations between KIAA0556 and MTs, MT-associated proteins (e.g., katanins) and IFT-B components.

A more favoured mechanistic scenario is that KIAA0556 regulates (ciliary) MT stability and/or dynamics. This is based on multiple lines of evidence. First, when overexpressed, human KIAA0556 co-localises with cytoplasmic MTs in hTERT-RPE1 cells, and the localisation of the nematode orthologue is suggestive of an association with the MT flares that exist at the base of sensory cilia [29]. Second, cytoplasmic MTs appear to be stabilised by artificial expression of high cytoplasmic levels of KIAA0556 in human cells. Third, human KIAA0556 binds MTs in vitro and biochemically associates with katanin subunits involved in MT severing and regulation; furthermore, KATNBL1 and KIAA0556 display overlapping subcellular localisation properties. Fourth, disruption of KIAA0556 reduces ciliary MT mass in nematodes and alters cilium formation and length in patient cells.

In one model, KIAA0556 would negatively regulate katanin function, which is proposed to sever cytoplasmic MT polymers in order to generate tubulin precursors for incorporation into the ciliary axoneme [45]. In support of this, *Tetrahymena* and mouse katanin negatively regulate MT acetylation and stability [21, 22, 48], whereas the opposite is observed for KIAA0556 (overexpression data). Also, *Tetrahymena*, *Chlamydomonas* and mammalian p60 and p80 katanins positively regulate cilium length [22, 45, 48], whereas KIAA0556 negatively regulates cilium elongation in KIAA0056 patient cells. On the other hand, a negative regulation model does not correlate with the reduced ciliogenesis potential observed for some KIAA0556 patient cells or explain why cilia have normal lengths in KIAA0556 disrupted mice and worms. However, similar ‘opposing’ observations have also been associated with katanins, which do not always positively regulate ciliogenesis. For example, p60 katanin overexpression (*Tetrahymena*, *Leishmania*, mouse) disrupts cilium formation and number, and loss of human KATNB1 results in supernumerary centrioles and cilia [21, 22, 43, 48]. Furthermore, katanins serve as negative regulators of MT mass in some contexts (e.g., *Tetrahymena*) and positive regulators in others (e.g., *C. elegans* meiotic cells and mammalian neurons) [22, 47, 65]. As suggested previously, these distinct regulatory outcomes for katanin may depend on the age, stability and subtype of the MTs being targeted, as well as the capacity of a particular cell type to initiate MT assembly from severed fragments of particular size [22]. Thus, as a potential katanin inhibitor, KIAA0556 might be expected to exert context-specific regulation of the MT cytoskeleton. Even within the same population of cells, subtle differences in MT state could tip the balance of katanin regulation by KIAA0556 towards net MT assembly or disassembly kinetics, which might explain why some KIAA0556 patient cells have long cilia, whereas others have no cilia. Indeed, it has been shown that mammalian cilia become abnormally long when cells are exposed to a relatively narrow nanomolar range of nocodazole, suggesting that cilium length regulation is strongly influenced by the concentration of free soluble tubulin [66].

The specific localisation of KIAA0056 and p60/80 katanins at the ciliary base [22, 43, 48, 67, 68] suggests that KIAA0056 regulates MT severing activities at this location. This is further supported by our finding that a *C. elegans* p80 KATNBL1 homologue, which binds KIAA0056 in human cells, is expressed almost exclusively in ciliated cells, thereby indicating a cilium-associated function. Although it remains to be shown how exactly this regulation would be achieved, binding of KIAA0556 to KATNBL1 could directly influence the MT binding or severing activities of katanin. Alternatively, the ability of KIAA0556 to associate with MTs could block access of katanin to its own MT binding sites.

Finally, it is interesting that the catalytic p60 katanin subunit *mei-1* is not expressed in all of the terminally differentiated neurons that express K04F10.2/KIAA0556. One possibility is that F47G4.5 serves as-of-yet unknown functions independent of the p60 enzymatic subunit of the katanin complex. Another possibility is that F47G4.5 and K04F10.2 tightly regulate the activity of very low levels of MEI-1 that might be present in many of these neurons. In *C. elegans*, *mei-1* is upregulated during meiosis, followed by a sharp downregulation during subsequent mitotic cycles. It is possible that p60 catalytic subunits turn over very slowly and persist in somatic cells at low levels, and that mechanisms involving F47G4.5/KATNBL1 and K04F10.2/KIAA0556 precisely regulate MT severing activities in post-mitotic ciliated neurons.

## Conclusions

We have identified a recessive *KIAA0556* nonsense mutation that causes a mild form of JBTS. We reveal that KIAA0556 is an evolutionarily conserved MT-binding protein enriched at the ciliary base, and provide evidence that KIAA0556 regulates MT stability, possibly via an interaction with katanin MT severing proteins. Future studies will be required to further investigate the pathogenic mechanisms of *KIAA0556*-related JBTS, including the full spectrum of phenotypes caused by mutations in this ciliary protein, as well as the precise role of KIAA0556 in ciliary MT regulation.

## Materials and methods

### Human subjects

Affected members were fully evaluated clinically and radiologically by board-certified clinical geneticists and paediatric neurologists. After obtaining informed consent from parents using a King Faisal Specialist Hospital and Research Center institutional review board-approved protocol (RAC# 2080006), blood was obtained from all members of the family in EDTA tubes for DNA extraction. Additional blood samples were obtained from the affected members for the establishment of Epstein-Barr virus-transformed lymphoblastoid cell lines. A small punch skin biopsy was also obtained from one affected member for the establishment of a primary fibroblast cell line.

### Gene mapping

Determination of the full set of autozygous intervals per genome (autozygome) was performed as described before. Briefly, DNA from each family member was run on the AxiomGWH SNP Chip platform following the manufacturer’s instructions (Affymetrix) for genome-wide genotyping. This was followed by the determination of autozygous intervals using runs of homozygosity (ROH) that were >2 Mb in size as surrogates of autozygosity using AutoSNPa software. Only autozygous intervals that were exclusively shared by the three affected members were considered candidate intervals to map the underlying mutation.

For whole-exome sequencing, 100 ng of DNA from the index case was treated to obtain the Ion Proton AmpliSeq library. Briefly, DNA was amplified in 12 separate wells using Exome Primer Pools, AmpliSeq HiFi mix (Thermo Fisher, Carlsbad, CA, USA) and ten amplification cycles. All 12 PCR pools were combined in one well and subjected to primer digestion, incubated with FuPa reagent (Thermo Fisher, Carlsbad, CA, USA). Amplified exome targets were ligated with Ion P1 and Ion Xpress Barcode adapters. After purification, libraries were quantified using quantitative PCR with the Ion Library Quantification Kit (Thermo Fisher, Carlsbad, CA, USA). The prepared exome library was further used for emulsion PCR on an Ion OneTouch System and templated Ion Sphere particles were enriched using Ion OneTouch ES, both procedures following the manufacturer’s instructions (Thermo Fisher, Carlsbad, CA, USA). The template-positive Ion PI Ion Sphere particles were processed for sequencing on the Ion Proton instrument (Thermo Fisher, Carlsbad, CA, USA). The total number of bases was 13.5 Gbp (79 million reads with median read length of 189 bp); 98 % of the bases (from 99.9 % of the reads) were aligned to the reference genome, achieving 93 % coverage of the target exonic regions at an average depth of 202×. Reads were mapped to UCSC hg19 (http://genome.ucsc.edu/) and variants identified using the Saudi Human Genome Program (SHGP) pipeline [69].

The analysis pipeline includes the following steps. First, the sequenced reads were examined for quality. Terminal regions of the reads with quality less than 20 were trimmed out. Second, the reads were aligned to the reference human genome (hg19) using the program tmap, distributed with the Torrent Suite package (https://github.com/iontorrent/TS). Third, the read alignments were processed to call the variants using the program TVC (Torrent Variant Caller). The output was a list of variants in the VCF file format. Fourth, the variants in the VCF files were annotated using an annotation pipeline including public and commercial knowledge databases in addition to an in-house database of Saudi mutations. The public databases include RefSeq, Online Mendelian Inheritance in Man (OMIM), GenBank, dbSNP, and 1000 Genomes, among others. In addition, the variants were annotated with pathogenicity scores, homozygosity/heterozygosity, and other parameters used to identify candidate causative variants. The damaging effect of a variant was predicted using PolyPhen-2 (http://genetics.bwh.harvard.edu/pph/), SIFT (http://sift.bii.a-star.edu.sg/), and Mutation Taster (http://www.mutationtaster.org/). The final step of the analysis pipeline is filtering, where only novel homozygous coding or splicing variants within the candidate autozygosity intervals are considered.

### Ciliogenesis assays

For analysis of ciliogenesis, patient and two control skin fibroblast cells were plated onto coverslips, cultured in Dulbecco’s modified Eagle’s medium (DMEM) supplemented with 10 % foetal bovine serum, glutamine, penicillin and streptomycin for 24 h. Subsequently, the medium was replaced with OptiMem (GIBCO) without serum and cultured for 72 h. Cells were fixed in 3.6 % paraformaldehyde for 10 min, then permeabilised with 0.1 % Triton X-100 (Sigma) followed by blocking in goat serum and staining with anti-acetylated alpha tubulin (SigmaT7451). Samples were washed with phosphate-buffered saline (PBS) and then incubated with fluorescent-dye-conjugated secondary antibody (ImmunoPure, Thermo Scientific). Coverslips were mounted in VectaShield containing DAPI (Vector Labs) and cells were observed under a fluorescent microscope (Nikon Eclipse 90i). For cilia forming frequency, more than 300 cells were counted for tubulin stained cilia. For ciliary length, over 100 tubulin-stained cilia from each cell line were assessed.

### Sequence and comparative structure analysis

Homologs of KIAA0556 were retrieved from an in-house protein sequence database of diverse eukaryotic species as previously described [70] using consecutively BLAST, PSI-BLAST [71], intermediate sequence searches (BLAST, but using increasingly divergent KIAA0556 homologs as query), and custom-built hidden Markov models (hmmer 3.0 [72]). From inspection of the sequences and distribution of sequences in eukaryotic species we can infer that all of these sequences are orthologous. The presence and absence of KIAA0556 orthologs was compared with the presence and absence of cilia. Sequences were aligned using MAFFT [73] (linsi --maxiterate 1000) and MUSCLE [70], and manually curated in JalView [74]. JalView was used for sequence visualisation. The internal repeats of human KIAA0556 and its orthologues were detected using RADAR [75], Internal Repeats Finder [76], Pfam [77], and SMART [78]. HHPred [24] was used to detect and evaluate the remote homology between the KIAA0556 repeats (including the permutated versions) and IFT25, taking into account position-specific information and structural features. To identify best-match circular permutations, repeat sequences were iteratively permutated (by transposing amino acids from the beginning of each repeat to the end of the repeat) and aligned against IFT25 using HHPred. Gaps were manually added to combine the pairwise alignments into a multiple sequence alignment.

### Generation of *Kiaa0556* genetrap mouse

The KIAA0556 genetrap mutant mice were generated through the UAB Transgenic/Embryonic Stem Cell Core Facility in the Hepatorenal Fibrocystic Disease Core Center using the BayGenomics D430042O09RikGt(RRG309)Byg ES cell line distributed by the Mutant Mouse Regional Resource Center (MMRRC). ES cells were injected into C57BL/6 blastocyst to generate chimeras. The chimeras were crossed to C57BL/6 mice for germ line passage of the mutant allele and to establish the mutant line.

### *C. elegans* strains

Strains were maintained at 15 °C or 20 °C using standard techniques [79]. Non-transgenic strains employed were N2 (Bristol), K04F10.2(*tm1830*), K04F10.2(*gk112869*), *arl-13(tm2322)*, F47G4.5(*ok2667*), *osm-5(p813)*, *klp-11(tm324)*, *mks-5(tm3100)*, *ifta-1(nx61)*, *nphp-4(tm925); him-5(e1490)*, and *che-11(e1810)*. Transgenic strains employed were N2;*oqEx[K04F10.2::GFP + unc-122p::DsRed*], N2;*oqEx[mei-1p::GFP + unc-122p::DsRed]*, N2;*oqEx[mei-2p::GFP + unc-122p::DsRed]*, N2;*oqEx[F47G4.4::GFP + unc-122p::DsRed]*, N2;*Ex[F47G4.5::GFP + unc-122p::DsRed]*, *lin-15(n765);kyIs104[str-1p::GFP + lin-15(+)]*, N2;*kyIs164[gcy-5p::GFP]*, N2;*myEx10[che-11::GFP + pRF4]*, N2;*lqIs2[osm-6::GFP]*, N2;*ejEx[osm-3::GFP + pRF4]*, N2;*Ex[K04F10.2::GFP + pRF4]* (injected at 5.0 ng/μl), N2;*Ex[K04F10.2:GFP + unc-22p::DsRed]* (injected at 0.5 ng/μl), *dpy-5(e907);Ex[F47G4.5::GFP + pCeh361 + unc-122p::DsRed]*, N2;*kyIs156[str-1p::odr-10::GFP]*, *dpy-5(e907); nxEx[bbs-7::GFP + pCeh361]*, N2;*oqEx58[arl-13::GFP + pRF4]*, N2;*Ex[mks-2::GFP + tram-1::tdTOMATO + pRF4]*, N2;*nxEx[mks-5::tdTOMATO + pRF4]*, and N2;*oqEx[arl-13::tdTOMATO + pRF4]*.

### *C. elegans* genetic crossing

Standard genetic crossing techniques were used to make double mutants and to introduce transgenes into genetic backgrounds. PCR genotyping was used to follow *tm1830*, *gk112869*, *tm2322*, *tm324*, *tm3100*, and *tm925* mutations. The dye-filling assay was employed to identify strains homozygous recessive for *p813*, *nx61*, and *e1810*.

### *C. elegans* fluorescence-tagged constructs

Constructs were generated by fusion PCR as previously described [80]. For the transcriptional GFP constructs, genomically amplified promoter sequences (including the first 13–20 bp of exon 1) for K04F10.2 (675 bp), *mei-1* (1033 bp), *mei-2* (527 bp), F47G4.4 (422 bp) and F47G4.5 (519 bp) were fused to GFP amplified from pPD95.67. For the K04F10.2 and F47G4.5 translational GFP constructs, the promoters plus the entire intronic and exonic sequences of these genes were genomically amplified and fused to GFP (amplified from pPD95.77). For the *arl-13p*::F47G4.5::GFP construct, the entire intronic and exonic sequence of F47G4.5 was first fused to GFP (from pPD95.77) and the resultant product fused to the promoter of *arl-13* (214 bp). To generate transgenic animals harbouring extrachromosomal arrays, all constructs were injected into N2 or *dpy-5(e905)* worms at 0.5–5 ng/μl (translational GFP constructs) or 50 ng/μl (transcriptional GFP constructs), together with *unc-122p::DsRed*, pCeh361, or pRF4 coinjection markers injected at 50 ng/μl.

### *C. elegans* behavioural and dye-uptake assays

For all assays, worm populations were synchronised by bleaching and early adult animals were assayed. For the osmotic avoidance assay, animals were transferred to the centre of a high concentration glycerol ring (8 M glycerol (Sigma), supplemented with Bromophenol Blue (Alfa Aesar)) on a non-seeded NGM plate and monitored for 10 minutes. Animals that had crossed the barrier were removed from the assay. The chemotaxis assays were performed as described previously [81]. Briefly, chemotactic attraction to benzaldehyde (Sigma) was assayed on 2 % minimal medium plates, supplemented with 5 % potassium phosphate pH 6.0, 1 mM CaCl_2_, and 1 mM MgSO_4_. Control and benzaldehyde spots were pipetted on either side of the plate and 50–100 animals placed at the centre of the plate. At 30 and 60 minutes animals at both spots were counted and the chemotaxis index (CI) calculated. For the dye-uptake assay, animals were incubated in fluorescent lipophilic DiI (Invitrogen; diluted 1:200 in M9 buffer) for 30 minutes and allowed to recover for 30 minutes on seeded NGM plates before imaging on a fluorescence microscope (Leica DM5000B). For the foraging assays, single worms were placed on an OP50 *Escherichia coli* seeded plate for 18 h and movement tracks scored with a grid reference to determine the extent of roaming [81].

### *C. elegans* fluorescence microscopy

Worms were immobilised with levamisole (Sigma; 40 mM in H_2_O) or polystyrene beads (0.1 μm; Polysciences) on 4 % or 10 % agarose pads, respectively. All GFP imaging, except for IFT assays, was performed on the Leica DM5000B fitted with epifluorescence. Images were acquired using a charge-coupled device camera (iXon + EM-CCD, Andor Technology), controlled by Andor Technology iQ 1.9 software. IFT assays were performed on a Nikon Eclipse Ti microscope, fitted with a 50 mW 488 nm laser, a CSU-X1 spinning disk unit (Yokogawa), a 100× 1.4NA Plan APO VC objective (Nikon) and a 1.5× Optovar optical zoom adaptor. Kymographs were generated and analysed in ImageJ 1.48 using the StackReg and TurboReg plugins, and in Icy (http://icy.bioimageanalysis.org/) using the Kymograph Tracker plugin to generate anterograde and retrograde kymographs [82].

### Mouse brain histology

Mouse brains were harvested and fixed in 4 % paraformaldehyde, embedded in OCT and frozen. The frozen brain tissue was sectioned 10 μm thick with a Thermo Cryostar NX50 cryostat and adhered to slides. Brain sections were permeabilised with 0.3 % Triton X-100 in PBS with 2 % donkey serum, 0.02 % sodium azide and 10 mg/ml bovine serum albumin (BSA). Primary antibody incubation with anti-adenylyl cyclase III (ACIII; 1:500; Santa Cruz Biotechnology, Santa Cruz, CA, USA) was performed for 16–24 h at 4 °C and secondary antibody incubation with Alexa Fluor-546 conjugated donkey anti-rabbit IgG (Invitrogen, Carlsbad, CA, USA) was performed for 1 h at room temperature. Nuclei were labelled with Hoechst (Sigma-Aldrich) and sections were mounted with DABCO (Sigma-Aldrich). A portion of the brain sections were also stained in 0.1 % cresyl violet (Nissl stain). Fluorescently labelled sections were imaged with a Hamamatsu C9100-50 EM-CCD camera (Hamamatsu Photonics K.K., Hamamatsu City, Japan) on an inverted Nikon TE2000-U microscope equipped with a 60× Plan Apochromat oil-immersion TIRFM objective (numerical aperture (NA), 1.49; Nikon Instruments Inc., Melville, NY, USA), and a Perkin Elmer Ultraview-ERS 6FE spinning disk confocal module controlled by Volocity 6.3 software (Perkin Elmer, Shelton, CT, USA). Alexa-594 conjugated antibody staining was imaged using a rhodamine/TRITC filter set (Chroma) and 20 mW 568 nm argon krypton laser (Melles Griot) and Hoechst staining using a 4′,6-diamidino-2-phenylindole (DAPI) filter set (Chroma) and 15 mW 405 nm diode laser (Qioptiq). Nissl stained sections and Evans blue injected brain slices were imaged using a Nikon SMZ800 dissecting scope with a Qimaging micropublisher 3.3RTV colour camera using Qcapture pro software (Qimaging, Canada).

### Ventricle dye injection

Evans blue (1 %; Sigma) in sterile PBS was injected into the ventricles of 8-week-old male mice. The mice, under anaesthesia by intraperitoneal injection of 2.5 % tribromoethanol (Sigma), were placed in a model 1900 stereotactic alignment system (Kopf Instruments, CA, USA). A hole was drilled 1 mm lateral and 0.3 mm posterior to the Bregma to target the lateral ventricle. Dye (2 μl) was slowly injected into the ventricle with a 10-μl Hamilton syringe that was inserted 2 mm into the brain. The syringe needle was removed 1 min after injection, the mice were killed by decapitation and the brains removed and sliced using a mouse brain slicing matrix block.

### High speed cilia imaging

Brains from knockout and wild-type mice were removed after anesthetization with isoflurane and decapitation. The ex vivo brains were sliced 400 μm thick with a Leica VT1000s vibrotome (Leica Instruments, Nussloch, Germany) on cover glass in room temperature sterile filtered, artificial cerebro-spinal fluid (125 mM NaCl, 2.5 mM KCl, 1.25 mM NaH2PO_4_, 2 mM CaCl_2_, 1 mM MgCl_2_, 25 mM NaHCO_3_, 25 mM glucose, pH 7.3) on the stage of a Nikon TE200 inverted microscope. Cilia motion in the ventricle was imaged with DIC illumination using a 100× objective (Nikon, plan-fluar 1.3NA) and a Cascade 1 K camera (Photometrics, Tucson, AZ, USA) restricted to a 50 × 50 pixel region to achieve 56 frames per second. Movies were analyzed in Metamorph 6.1 (Molecular Devices, CA, USA) to extract intensity changes over time of a fixed spot as a cilium swept past. A fast Fourier transform of these data were performed in Excel (Microsoft, WA, USA) to determine the frequency of cilia oscillation.

### Bead flow imaging

Brains from knockout and wild-type mice were removed after anaesthetization with isoflurane and decapitation. The ex vivo brains were cut sagittally down the midline and placed cut surface up on an upright Zeiss Axioskop microscope (Carl Zeiss Microscopy, LLC) immersed in room-temperature artificial cerebro-spinal fluid. Fluorescent red beads (carboxylate modified polystyrene latex, Sigma) were applied to the ventricle surface at 0.25 % suspension. Bead motility within the ventricle was imaged using a Hitachi KP-M2RN CCD camera (Hitachi Kokusai Electric Inc.) with a 10× air objective (Zeiss plan-neofluar 0.3NA). Bead velocity was tracked and analyzed using ImageJ (National Institutes of Health) using the trackmate plugin.

### Human cDNA plasmids

Plasmids encoding full-length KIAA556 (RefSeq accession numbers NM_015202.2 [gene], NP_056017.2 [protein]) and full-length KATNBL1 (RefSeq accession numbers NM_024713.2 [gene], NP_078989 [protein]) and fragments thereof were generated by Gateway-adapted PCR and subsequently cloned using Gateway cloning Technology (Life Technologies) according to the manufacturer's instructions. We generated plasmids encoding GFP-KIAA0556, mRFP-KIAA0556, GFP-KATNBL1 and mRFP-KATNBL1 for localisation studies, SF-TAP-KIAA0556 for localization and tandem affinity purification experiments and PalMyr-CFP-KIAA0556 and PalMyr-CFP-KATNBL1 for colocalisation experiments. Sequences of all entry clones were verified by Sanger sequencing.

### Microtubule binding assay

Binding of KIAA556 to MTs was tested using a spin down assay kit (Cytoskeleton Inc., Denver) as previously described [83]. GFP-KIAA0556 or GFP were expressed in HEK293 cells. Microtubules were polymerised according to the user’s manual and incubated with 10 μl of total cell lysate of HEK293 cells expressing GFP-KIAA0556 or GFP at room temperature for 30 min. After centrifugation at 100,000 g for 45 min in Beckman Coultor Optima MAX ultracentrifuge (Krefeld, Germany), supernatants and pellets were analyzed by immunoblotting using anti-GFP antibodies (ab6556, Abcam, Cambridge, UK).

### Yeast two-hybrid assay

The GAL4-based yeast two-hybrid system (HybriZAP, Stratagene) was used for identifying binary protein–protein interaction partners of KIAA0556. We have cloned several fragments of KIAA0556 (Additional file 7), containing one or more of the predicted repeat sequences, to which we fused either the DNA binding domain (GAL4-BD) or the activation domain (GAL4-AD). In yeast cells, constructs were transformed in as previously described [84]. Yeast strains PJ69-4A (GAL4-BD) and PJ69-4α (GAL4-AD), both of which carry the HIS3 (histidine), ADE2 (adenine), and LacZ (b-galactosidase) reporter genes, were used as a hosts. GAL4-BD constructs were tested for autoactivation on selective growth media. Via mating with the reciprocal yeast strain, KIAA0556 constructs were used as a bait to test the interaction with >200 previously described ciliopathy and cilium-associated proteins. Interactions were analyzed by assessment of reporter gene (HIS3 and ADE2) activation via growth on selective media and b-galactosidase colorimetric filter lift assays (LacZ reporter gene). cDNA inserts of clones containing putative interaction partners were confirmed by Sanger sequencing. Putative interaction partners were confirmed by a dedicated one-on-one interaction assay in yeast strain PJ69-4A.

### hTERT-RPE1 cell transfection and imaging protocols

Human TERT-immortalised retinal pigment epithelium 1 (hTERT- RPE1) cells were cultured as previously described [84]. Cells were seeded on coverslips, grown to 80 % confluency, and subsequently serum starved for 24 h in medium containing only 0.2 % foetal calf serum for inducing cilium growth. The cells were then (co)-transfected with the various expression constructs using Lipofectamine 2000 (Life Technologies) according to the manufacturer’s instructions. For MT stabilization assays, one day after transfection, cells were treated for 10 minutes with DMSO (controls) or nocodazole (10 μM). Cells were subsequently fixed in 2 % paraformaldehyde for 20 min, treated with 1 % Triton X-100 in PBS for 5 min, and blocked in 2 % BSA in PBS for 20 min. Cells were incubated with primary antibodies: GT335 (1:500; gift from C. Janke, Institut Curie, France), anti-FLAG (1:500; Rabbit polyclonal, Sigma Aldrich), anti-RPGRIP1L SNC040 (1:500; Arts et al. [38]), anti-acetylated tubulin (1:1000; Sigma Aldrich) diluted in 2 % BSA in PBS, for 1 h. After washing in PBS, the cells were incubated with the secondary antibody for 45 min. Secondary antibodies, goat anti-mouse, goat anti-guinea pig, and goat anti-rabbit (Alexa 488, 568, and 647, respectively; 1:500; Life Technologies) were diluted in 2 % BSA in PBS. Cells were washed three times with PBS and once briefly with milliQ water before being mounted in Vectashield containing DAPI (Vector Laboratories). The cellular localization was analyzed with a Zeiss Axio Imager Z2 fluorescence microscope. Optical sections were generated through structured illumination by the insertion of an ApoTome slider into the illumination path and subsequent processing with AxioVision (Zeiss) software. Confocal laser scanning microscopy was performed using Leica’s DM IRE2 TCS SP2 AOBS. Maximum projections were generated and subsequently processed using Photoshop CS6 (Adobe Systems).

### TAP of protein complexes

HEK293T were cultured in high glucose DMEM AQmedia (Sigma Aldrich), supplemented with 10 % foetal calf serum, 1 % penicillin/streptomycin and 1 mM sodium pyruvate. For DNA transfections, HEK293T cells were seeded, grown overnight, and then transfected using polyethylenimine transfection. Cells transiently expressing the Streptavidin-FLAG (SF-TAP)-tagged KIAA0556 fusion protein were lysed in lysis buffer containing 0.5 % Nonidet-P40, protease inhibitor cocktail (Roche), and phosphatase inhibitor cocktails I and II (Sigma-Aldrich) in Tris-buffered saline (30 mM Tris-HCl, pH 7.4, and 150 mM NaCl) for 20 minutes at 4 °C. The streptavidin- and FLAG-based tandem affinity purification steps were performed as previously described [42, 85]. Five percent of the final eluate was evaluated by SDS-PAGE followed by silver staining, according to standard protocols, while the remaining 95 % was subjected to protein precipitation with chloroform and methanol. Protein precipitates were subsequently subjected to mass spectrometry analysis and peptide identification as previously described [86]. Proteins identified in >1 out of 17 SF-TAP control experiments (empty vector) were removed.

### Ethics approval

The experimental methods of the study are in concordance with the Helsinki Declaration. All human subjects in the study have provided written informed consent for research and publication as part of an institutional review board-approved protocol (KFSRHC RAC# 2080006). All mice were maintained on an inbred C57BL/6 genetic background and experimental procedures were approved by the Institutional Animal Care and Use Committee (IACUC) regulations at the University of Alabama at Birmingham under animal protocol number (141109276).

### Availability of data and material

A link to the VCF file of the exome performed on the index will be provided upon request. The data set of fluorescence images and movies that support the results of this article is available in the Zenodo repository, DOI accession 10.5281/zenodo.33941 (deposited 18-11-2015), and accessed at https://zenodo.org/record/33941 [87].

## Additional files

Additional file 1:
**The phylogenetic distribution and sequence conservation of KIAA0556 orthologs in eukaryotes.** Presence and sequence conservation of KIAA0556 are projected on the eukaryotic species tree to visualise the phylogenetic distribution of KIAA0556 orthologues as well as the distribution of the triple-repeat and quadruple-repeat configurations of the DUF4457 domains of unknown function. The *black circles* and *white circles* indicate which eukaryotic species contain or lack cilia/flagella. Recent KIAA0556 duplicates in *Branchiostoma floridae* and *Paramecium tetraurelia* are denoted by *x2*. **Dictyostelium discoideum* protein sequence contains many “N”s (uncalled bases) in the N-terminal part of the sequence, indicative of sequencing errors. As a result we are unable to identify whether this region is indeed homologous to the human KIAA0556 N-terminus. Boxed schematic at bottom shows examples of *C. elegans* and human KIAA0556 with three and four repeat domains, respectively. (PDF 283 kb) Additional file 2:
**Alignment of IFT25 with permutated KIAA0556 repeat sequences.** When aligned using HHpred, a significant part of the *Chlamydomonas* IFT25 N-terminus was unmatched with human KIAA0556 and significant sequence remained at the C-terminus of the repeats, suggesting a circular permutation relationship between the repeats and IFT25. Shown is a HHpred alignment of IFT25 orthologues with permutated repeat sequences (r1–4) from KIAA0556 orthologues, which results in improved sequence matches. In each permutated repeat sequence, 30–40 amino acids from the beginning of each repeat have been added to the end of the same repeat (denoted by *red box*) using manual editing. The precise number of amino acids transposed in this way was calculated by iterative comparison. Alignment was edited manually to improve gapped regions and other minor adjustments. Attributes of the amino acids are coloured using ClustalX colour schemes. *light blue* = hydrophobic; *cyan* = aromatic; *green* = polar; *red* = positive charge; *magenta* = negative charge; *orange* = glycine; *light green* = proline. Positions are only coloured if 25 % or more of the residues share a given property. Secondary structures of IFT25 are shown in the first row; *arrows* (*yellow*) denote beta strands; *cylinder* (*blue*) denotes helical structure. (JPG 1555 kb) Additional file 3:
**Ciliary phenotypes that are unaffected in** ***C. elegans*** **K04F10.2(** ***tm1830*** **) mutants.** **a** K04F10.2 mutants possess normal fluorescent dye (DiI) filling in amphid (head) and phasmid (tail) neurons. Scale bars, 15 μm. **b** The lengths and morphologies of various sensory neuronal cilia are normal in K04F10.2 mutants. Shown are fluorescence images of cilia from worms expressing *str-1p::*GFP (AWB neuron), *gcy-5p::*GFP (ASER neuron) and OSM-6::GFP (PHA/B neurons) transgenes. Numbers (± standard error of the mean) refer to cilium lengths. Scale bars, 2 μm. **c–e** K04F10.2 mutants possess normal sensory benzaldehyde chemoattraction (n = 10), osmotic avoidance (n = 10), and foraging/roaming (n = 34) behaviours. *che-11(e1810)* and *osm-5(p813)* are negative controls. **p* < 0.05 (t-test versus wild type (*WT*)). (JPG 696 kb) Additional file 4:
**IFT analysis in** ***C. elegans*** **K04F10.2(** ***tm1830*** **) mutants.** **a** Intraflagellar transport rates in wild-type and K04F10.2(*tm1830*) mutant worms. Shown are the anterograde and retrograde velocities (μm.s^-1^/standard deviation (SD)) of GFP-tagged IFT proteins along amphid and phasmid channel cilia (combined; *top rows*), or phasmid cilia only (*bottom rows*). *t-test* pairwise comparison with wild-type controls, *n* number of particles, *N* measured number of amphids and phasmids. OSM-3 is the worm orthologue of KIF17; CHE-11 is the worm orthologue of IFT140; OSM-6 is the worm orthologue of IFT52. **b** Representative fluorescence images of phasmid cilia showing normal IFT protein localisations and distributions in *tm1830* mutants. *ds* distal segment, *ms* middle segment, *bb* basal body region, *den* dendrite. All images are similarly scaled and orientated (*arrow* denotes basal body). Scale bar, 3 μm. **c** Representative kymographs (time (t) over distance (d) plots) used to generate IFT rate measurements. For each kymograph, the horizontal axis (distance) is 5 μm and the vertical axis (time) is 25 seconds. **d** Distribution plots of IFT protein velocities. (JPG 1951 kb) Additional file 5:
**Data supplementary to the nocodazole destabilization assay shown in Fig.** **7** **. a, b** **Replicate images of DMSO or nocodazole-treated hTERT-RPE1 cells.** Cells were transfected with SF-TAP-tagged KIAA0556 (detected with anti-FLAG immunostaining; *green*) or GFP-KIAA0556 and counterstained with anti-acetylated tubulin (*red*) and DAPI (*blue*). Cells with high KIAA0556 expression are characterised by a filamentous staining pattern and spots of accumulated KIAA0556 signal. In non-transfected cells, 10 minute nocodazole treatment resulted in the loss of a stabilised MT network (see especially the high exposure images), as judged by loss of (almost) all cytoplasmic acetylated tubulin staining and/or the absence of a filamentous staining pattern. In transfected cells (expressing KIAA0556), a filamentous acetylated tubulin staining pattern remained. See also Fig. 7 for examples. Scale bar, 20 μm. **c** Quantification of the presence of a detectable filamentous acetylated alpha tubulin MT network in GFP-KIAA0556 transfected and non-transfected cells, treated with DMSO or 10 μM nocodazole for 10 minutes. MT networks could be identified in approximately 80 % of GFP-KIAA0556 transfected cells (n = 15) compared with untransfected cells (n > 200). Due to the lower expression level and transfection efficiency of GFP-KIAA0556 (compared with SF-TAP-tagged KIAA0556 in Fig. 7c), only a relatively small number of transfected cells could be analysed. (JPG 1596 kb) Additional file 6:
**Results of the SF-TAP analysis with over-expressed N-terminally SF-TAP-tagged KIAA0556 in HEK293T cells.** Shown is the number of unique identified peptides as well as the sequence coverage for each protein detected by mass spectrometry. Proteins identified in >1 out 17 SF-TAP control experiments (empty vector) were removed. (XLSX 28 kb) Additional file 7:
**Supplementary information to the data in Fig.** **8** **. a** **Schematic representation of all the different KIAA0556 fragments used to screen our selection of 200+ ciliary proteins.** The predicted protein repeat domains, shown in Additional files 1 and 2, are depicted as d1 to d4. Constructs were generated containing isolated domains as well as a combination of domains. **b** Single transfections of PalMyr-KIAA0556 and mRFP-KATNBL1, showing that membrane localisation of the mRFP tagged protein is indeed dependent on the interaction with the PalMyr-tagged protein. (JPG 491 kb) Additional file 8:
**Post-embryonic tissue expression of** ***C. elegans*** **katanin genes** ***mei-1*** **,** ***mei-2*** **and F47G4.4.** Shown are fluorescence images of worms expressing a transcriptional GFP reporter under the control of the indicated gene’s promoter, which stains the entire cell in which it is expressed. DiI (*red*) co-stain identifies six pairs of ciliated amphid neurons and both pairs of ciliated phasmid neurons. *Arrowheads* denote cells with both red and green signals. Other ciliated head cells are identifiable by long dendritic processes (*arrows*) extending to the anterior end of the worm. Scale bars, 20 μm (all images similarly scaled). (JPG 611 kb)

## Abbreviations

bp: Base pair
BSA: Bovine serum albumin
DMEM: Dulbecco’s modified Eagle’s medium
ES: Embryonic stem
GFP: Green fluorescent protein
IFT: Intraflagellar transport
JBTS: Joubert syndrome
MKS: Meckel Gruber syndrome
mRFP: Monomeric red fluorescent protein
MRI: Magnetic resonance imaging
MT: Microtubule
NA: Numerical aperture
NPHP: Nephronophthisis
OFD: Oral-facial digital syndrome
PCR: Polymerase chain reaction
SF: Strep-Flag
TAP: Tandem affinity purification
