# Supplementary figures and images for: KIAA0556 is a novel ciliary basal body component mutated in Joubert syndrome
Source: Genome Biol. 2015 Dec 29;16:293. doi: 10.1186/s13059-015-0858-z (PMC4699358; doi:10.1186/s13059-015-0858-z)

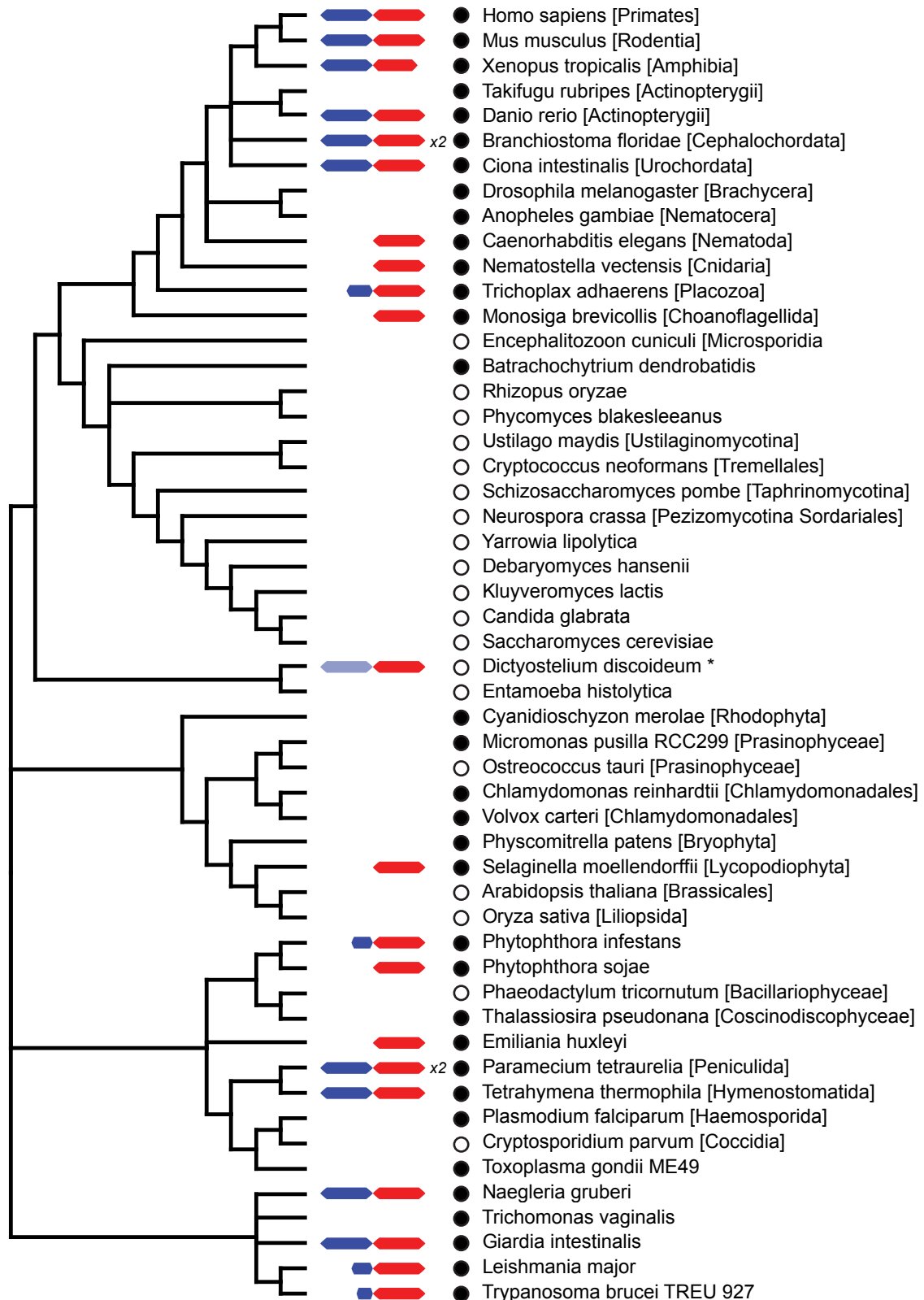

Supplement: Additional file 1: — The phylogenetic distribution and sequence conservation of KIAA0556 orthologs in eukaryotes. Presence and sequence conservation of KIAA0556 are projected on the eukaryotic species tree to visualise the phylogenetic distribution of KIAA0556 orthologues as well as the distribution of the triple-repeat and quadruple-repeat configurations of the DUF4457 domains of unknown function. The black circles and white circles indicate which eukaryotic species contain or lack cilia/flagella. Recent KIAA0556 duplicates in Branchiostoma floridae and Paramecium tetraurelia are denoted by x2. *Dictyostelium discoideum protein sequence contains many “N”s (uncalled bases) in the N-terminal part of the sequence, indicative of sequencing errors. As a result we are unable to identify whether this region is indeed homologous to the human KIAA0556 N-terminus. Boxed schematic at bottom shows examples of C. elegans and human KIAA0556 with three and four repeat domains, respectively. (PDF 283 kb) [file 13059_2015_858_MOESM1_ESM.pdf]

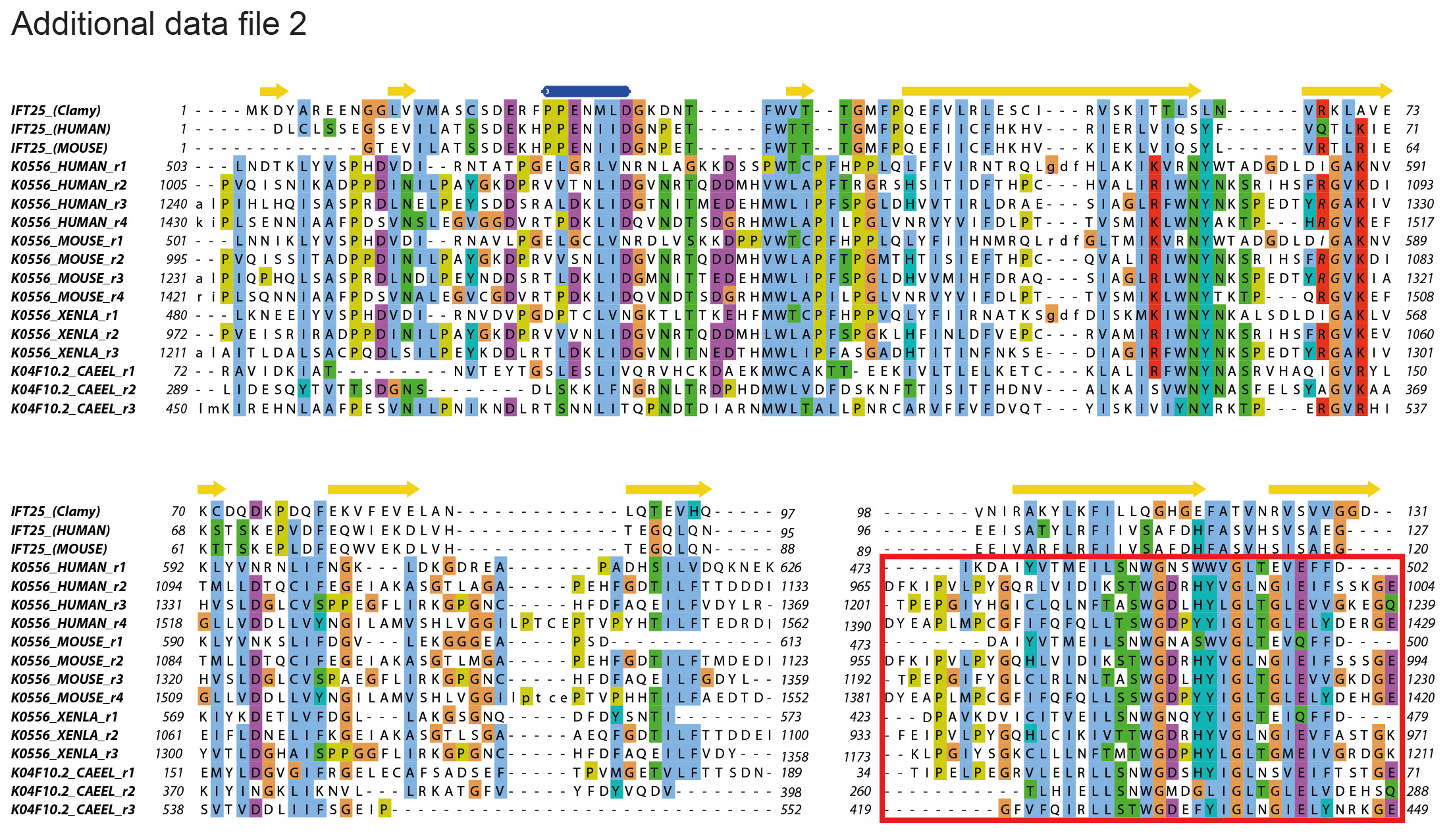

Supplement: Additional file 2: — Alignment of IFT25 with permutated KIAA0556 repeat sequences. When aligned using HHpred, a significant part of the Chlamydomonas IFT25 N-terminus was unmatched with human KIAA0556 and significant sequence remained at the C-terminus of the repeats, suggesting a circular permutation relationship between the repeats and IFT25. Shown is a HHpred alignment of IFT25 orthologues with permutated repeat sequences (r1–4) from KIAA0556 orthologues, which results in improved sequence matches. In each permutated repeat sequence, 30–40 amino acids from the beginning of each repeat have been added to the end of the same repeat (denoted by red box) using manual editing. The precise number of amino acids transposed in this way was calculated by iterative comparison. Alignment was edited manually to improve gapped regions and other minor adjustments. Attributes of the amino acids are coloured using ClustalX colour schemes. light blue = hydrophobic; cyan = aromatic; green = polar; red = positive charge; magenta = negative charge; orange = glycine; light green = proline. Positions are only coloured if 25 % or more of the residues share a given property. Secondary structures of IFT25 are shown in the first row; arrows (yellow) denote beta strands; cylinder (blue) denotes helical structure. (JPG 1555 kb) [file 13059_2015_858_MOESM2_ESM.jpg]

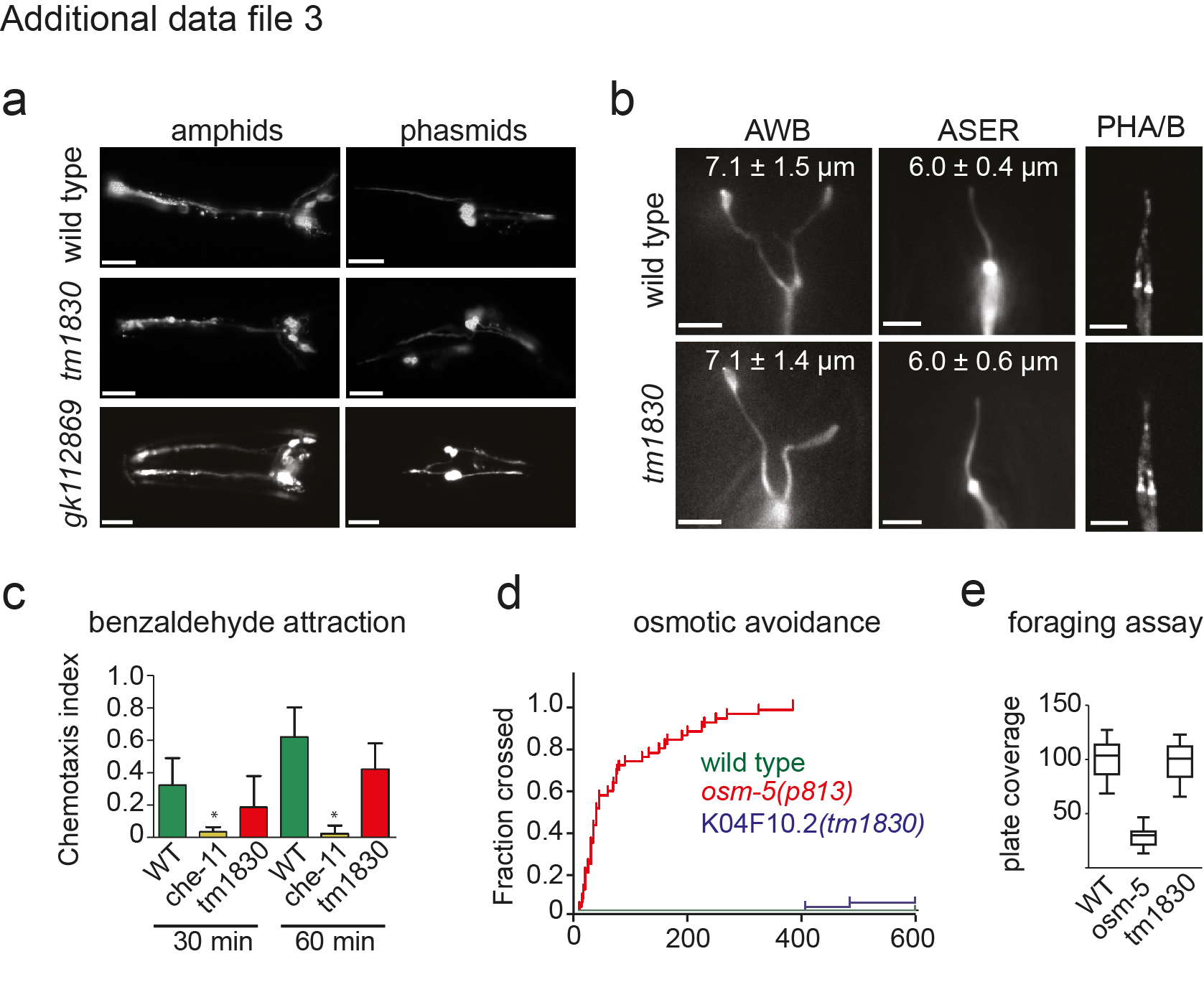

Supplement: Additional file 3: — Ciliary phenotypes that are unaffected in C. elegans K04F10.2( tm1830 ) mutants. a K04F10.2 mutants possess normal fluorescent dye (DiI) filling in amphid (head) and phasmid (tail) neurons. Scale bars, 15 μm. b The lengths and morphologies of various sensory neuronal cilia are normal in K04F10.2 mutants. Shown are fluorescence images of cilia from worms expressing str-1p::GFP (AWB neuron), gcy-5p::GFP (ASER neuron) and OSM-6::GFP (PHA/B neurons) transgenes. Numbers (± standard error of the mean) refer to cilium lengths. Scale bars, 2 μm. c–e K04F10.2 mutants possess normal sensory benzaldehyde chemoattraction (n = 10), osmotic avoidance (n = 10), and foraging/roaming (n = 34) behaviours. che-11(e1810) and osm-5(p813) are negative controls. *p < 0.05 (t-test versus wild type (WT)). (JPG 696 kb) [file 13059_2015_858_MOESM3_ESM.jpg]

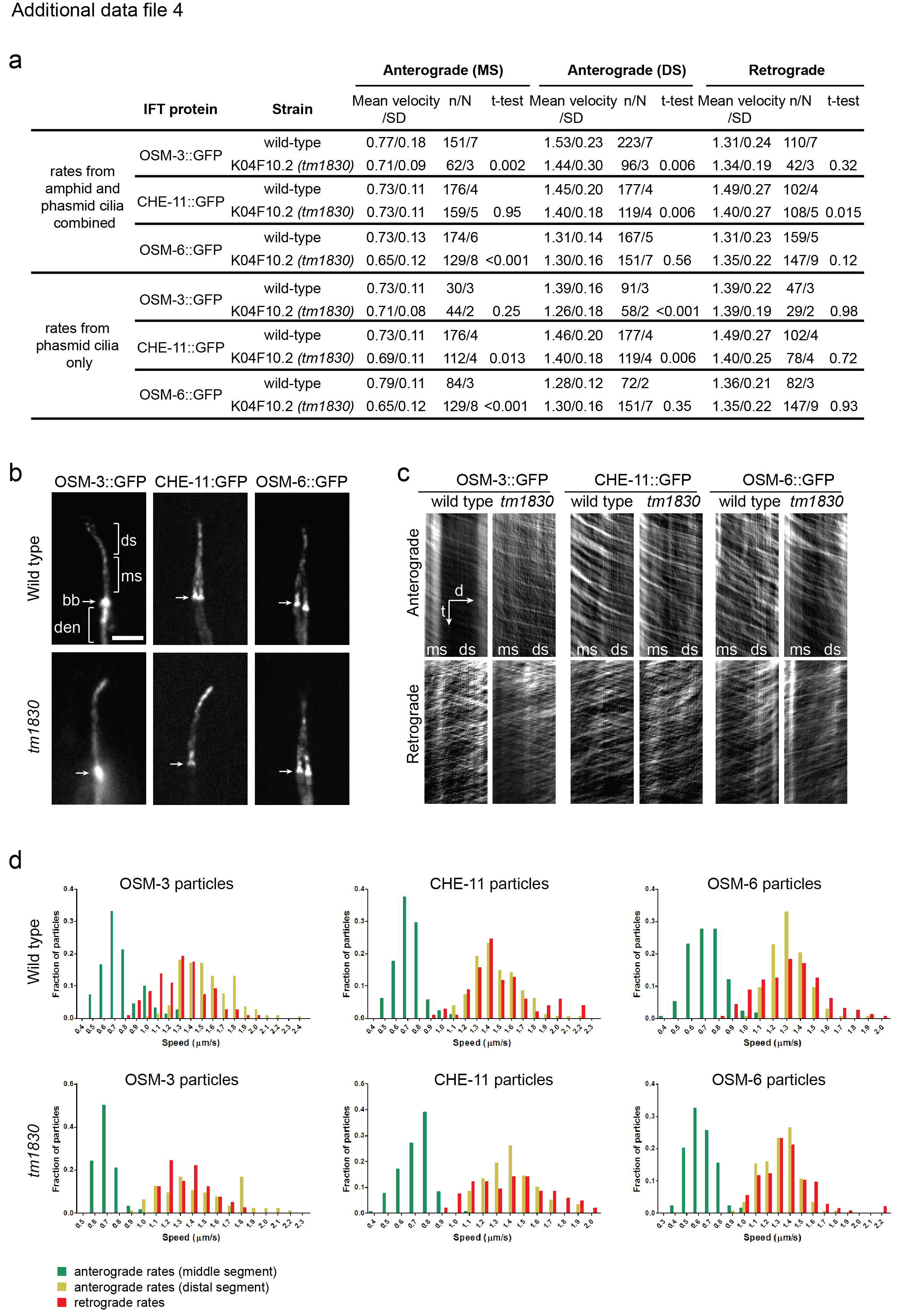

Supplement: Additional file 4: — IFT analysis in C. elegans K04F10.2( tm1830 ) mutants. a Intraflagellar transport rates in wild-type and K04F10.2(tm1830) mutant worms. Shown are the anterograde and retrograde velocities (μm.s-1/standard deviation (SD)) of GFP-tagged IFT proteins along amphid and phasmid channel cilia (combined; top rows), or phasmid cilia only (bottom rows). t-test pairwise comparison with wild-type controls, n number of particles, N measured number of amphids and phasmids. OSM-3 is the worm orthologue of KIF17; CHE-11 is the worm orthologue of IFT140; OSM-6 is the worm orthologue of IFT52. b Representative fluorescence images of phasmid cilia showing normal IFT protein localisations and distributions in tm1830 mutants. ds distal segment, ms middle segment, bb basal body region, den dendrite. All images are similarly scaled and orientated (arrow denotes basal body). Scale bar, 3 μm. c Representative kymographs (time (t) over distance (d) plots) used to generate IFT rate measurements. For each kymograph, the horizontal axis (distance) is 5 μm and the vertical axis (time) is 25 seconds. d Distribution plots of IFT protein velocities. (JPG 1951 kb) [file 13059_2015_858_MOESM4_ESM.jpg]

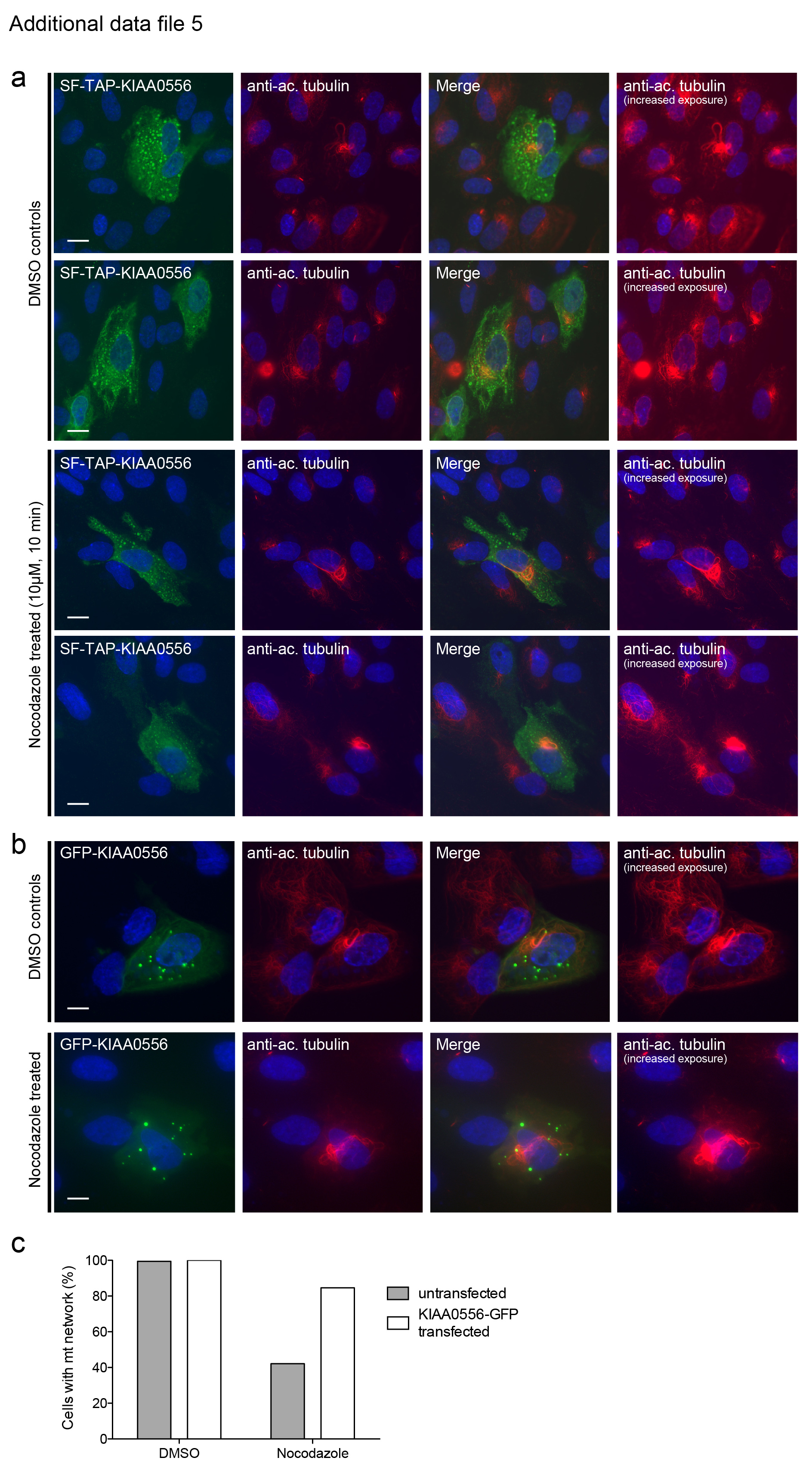

Supplement: Additional file 5: — Data supplementary to the nocodazole destabilization assay shown in Fig. 7 . a, b Replicate images of DMSO or nocodazole-treated hTERT-RPE1 cells. Cells were transfected with SF-TAP-tagged KIAA0556 (detected with anti-FLAG immunostaining; green) or GFP-KIAA0556 and counterstained with anti-acetylated tubulin (red) and DAPI (blue). Cells with high KIAA0556 expression are characterised by a filamentous staining pattern and spots of accumulated KIAA0556 signal. In non-transfected cells, 10 minute nocodazole treatment resulted in the loss of a stabilised MT network (see especially the high exposure images), as judged by loss of (almost) all cytoplasmic acetylated tubulin staining and/or the absence of a filamentous staining pattern. In transfected cells (expressing KIAA0556), a filamentous acetylated tubulin staining pattern remained. See also Fig. 7 for examples. Scale bar, 20 μm. c Quantification of the presence of a detectable filamentous acetylated alpha tubulin MT network in GFP-KIAA0556 transfected and non-transfected cells, treated with DMSO or 10 μM nocodazole for 10 minutes. MT networks could be identified in approximately 80 % of GFP-KIAA0556 transfected cells (n = 15) compared with untransfected cells (n > 200). Due to the lower expression level and transfection efficiency of GFP-KIAA0556 (compared with SF-TAP-tagged KIAA0556 in Fig. 7c), only a relatively small number of transfected cells could be analysed. (JPG 1596 kb) [file 13059_2015_858_MOESM5_ESM.jpg]

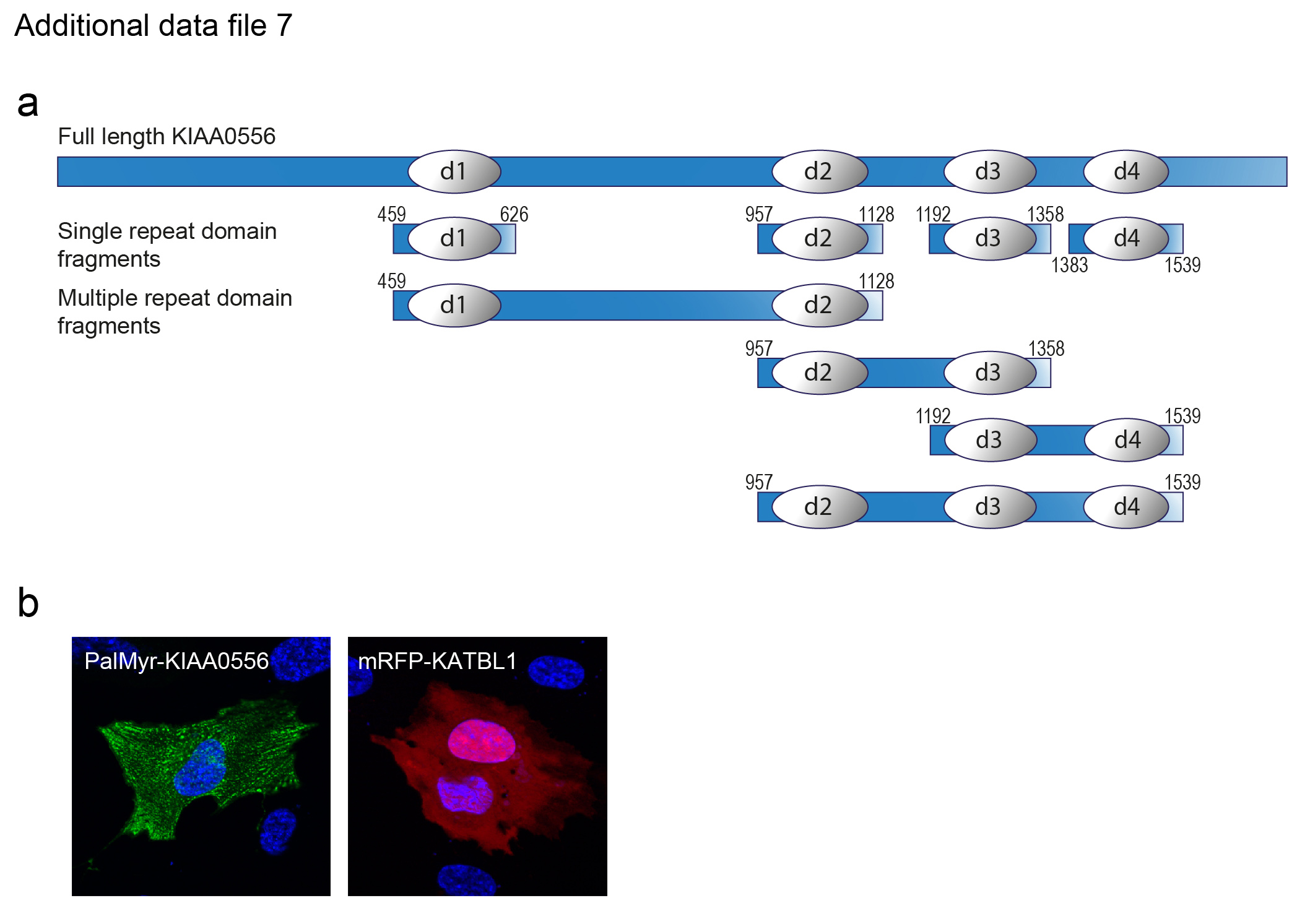

Supplement: Additional file 7: — Supplementary information to the data in Fig. 8 . a Schematic representation of all the different KIAA0556 fragments used to screen our selection of 200+ ciliary proteins. The predicted protein repeat domains, shown in Additional files 1 and 2, are depicted as d1 to d4. Constructs were generated containing isolated domains as well as a combination of domains. b Single transfections of PalMyr-KIAA0556 and mRFP-KATNBL1, showing that membrane localisation of the mRFP tagged protein is indeed dependent on the interaction with the PalMyr-tagged protein. (JPG 491 kb) [file 13059_2015_858_MOESM7_ESM.jpg]

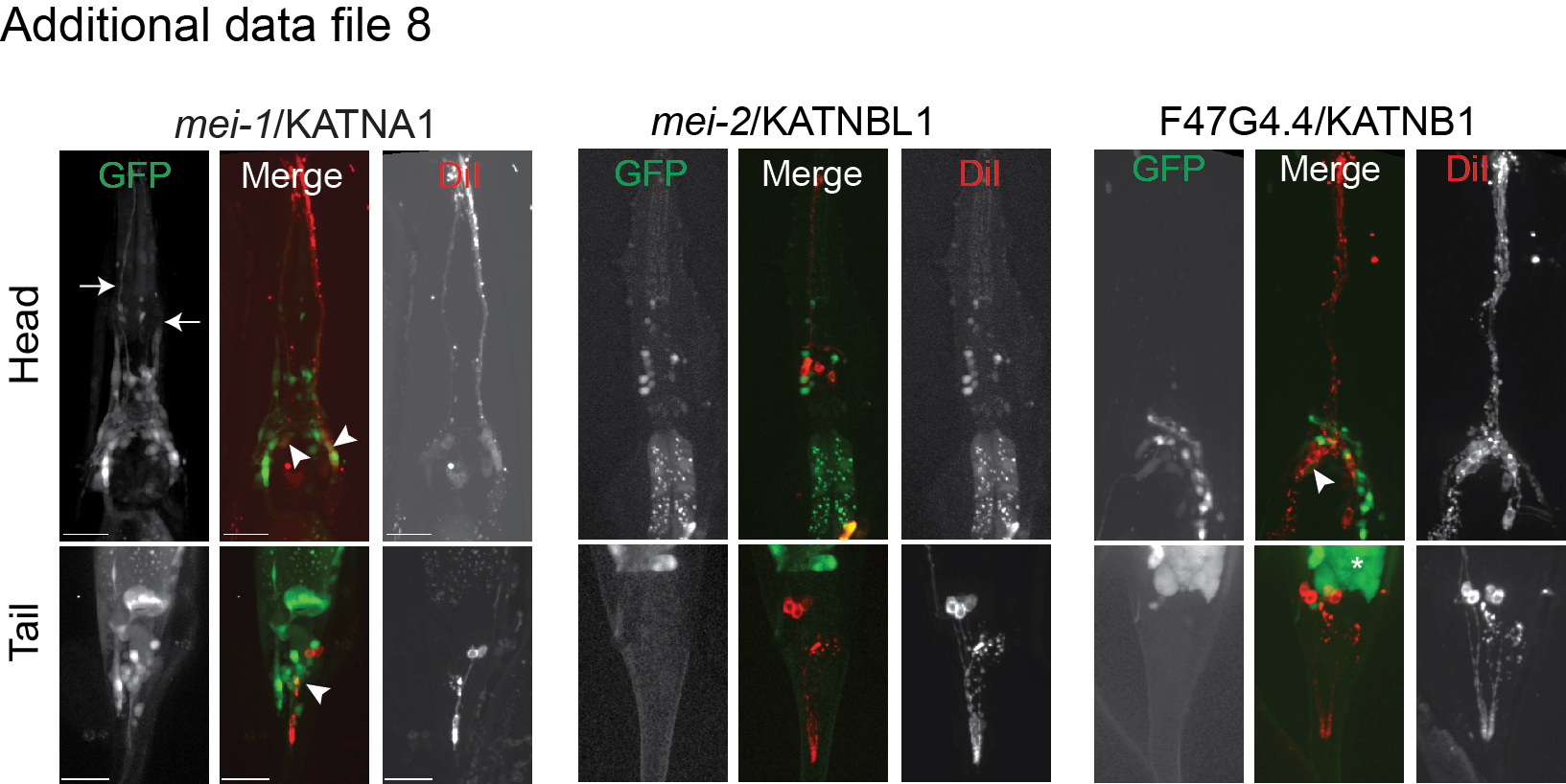

Supplement: Additional file 8: — Post-embryonic tissue expression of C. elegans katanin genes mei-1 , mei-2 and F47G4.4. Shown are fluorescence images of worms expressing a transcriptional GFP reporter under the control of the indicated gene’s promoter, which stains the entire cell in which it is expressed. DiI (red) co-stain identifies six pairs of ciliated amphid neurons and both pairs of ciliated phasmid neurons. Arrowheads denote cells with both red and green signals. Other ciliated head cells are identifiable by long dendritic processes (arrows) extending to the anterior end of the worm. Scale bars, 20 μm (all images similarly scaled). (JPG 611 kb) [file 13059_2015_858_MOESM8_ESM.jpg]
